# Integrative modeling uncovers p21-driven drug resistance and prioritizes therapies for PIK3CA-mutant breast cancer

**DOI:** 10.1038/s41698-024-00496-y

**Published:** 2024-01-26

**Authors:** Hon Yan Kelvin Yip, Sung-Young Shin, Annabel Chee, Ching-Seng Ang, Fernando J. Rossello, Lee Hwa Wong, Lan K. Nguyen, Antonella Papa

**Affiliations:** 1 Cancer Program, Monash Biomedicine Discovery Institute and Department of Biochemistry and Molecular Biology, Monash University, Melbourne, VIC 3800 Australia; 2 Bio21 Mass Spectrometry and Proteomics Facility, The University of Melbourne, Parkville, VIC 3010 Australia; 3 Murdoch Children’s Research Institute, The Royal Children’s Hospital, Melbourne, VIC 3052 Australia; 4 Novo Nordisk Foundation Center for Stem Cell Medicine, Murdoch Children’s Research Institute, Melbourne, VIC 3052 Australia; 5 Department of Clinical Pathology, University of Melbourne, Melbourne, VIC Australia; 6 Australian Regenerative Medicine Institute, Monash University, Melbourne, VIC Australia; 7 Present Address: Centre for Muscle Research, Department of Anatomy and Physiology, The University of Melbourne, Melbourne, VIC 3010 Australia

**Keywords:** Systems biology, Predictive markers, Senescence, Phosphoinositol signalling

## Abstract

Utility of PI3Kα inhibitors like BYL719 is limited by the acquisition of genetic and non-genetic mechanisms of resistance which cause disease recurrence. Several combination therapies based on PI3K inhibition have been proposed, but a way to systematically prioritize them for breast cancer treatment is still missing. By integrating published and in-house studies, we have developed in silico models that quantitatively capture dynamics of PI3K signaling at the network-level under a BYL719-sensitive versus BYL719 resistant-cell state. Computational predictions show that signal rewiring to alternative components of the PI3K pathway promote resistance to BYL719 and identify PDK1 as the most effective co-target with PI3Kα rescuing sensitivity of resistant cells to BYL719. To explore whether PI3K pathway-independent mechanisms further contribute to BYL719 resistance, we performed phosphoproteomics and found that selection of high levels of the cell cycle regulator p21 unexpectedly promoted drug resistance in T47D cells. Functionally, high p21 levels favored repair of BYL719-induced DNA damage and bypass of the associated cellular senescence. Importantly, targeted inhibition of the check-point inhibitor CHK1 with MK-8776 effectively caused death of p21-high T47D cells, thus establishing a new vulnerability of BYL719-resistant breast cancer cells. Together, our integrated studies uncover hidden molecular mediators causing resistance to PI3Kα inhibition and provide a framework to prioritize combination therapies for PI3K-mutant breast cancer.

## Introduction

**Fig. 1:**
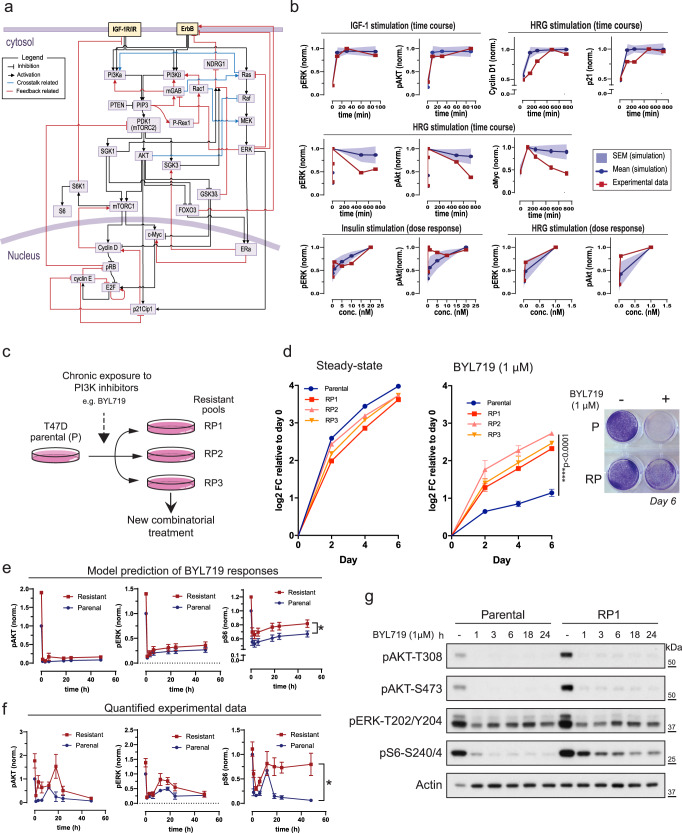
Mathematical modeling captures signaling pathways behaviors. **a** Integrated PI3K network model. IGFR: insulin-like growth factor-1 (IGF-1) receptor; IR insulin receptor, ErbB ErbB receptor family. Bar-headed lines indicate inhibitory processes, arrows reflect activations. Blue lines denote crosstalk reactions, red lines reflect feedback reactions. See also Supplementary Tables 1 and 2. **b** Comparison of model predictions (blue lines) and experimental data (red lines) in time-course and dose-response experiments with indicated time and growth factors’ concentrations. Solid lines indicate mean values shown with associated standard error (*n* = 77 best-fitted parameter sets). ERK1/2 and AKT phosphorylation were analyzed at 10, 30 and 90 min of IGF-1 (13 nM) stimulation. Data were downloaded from HMS LINCS Center (https://www.cancerbrowser.org/). Expression of Cyclin D1, p21, and cMyc, ERK1/2 and AKT phosphorylation were analyzed at 120, 240, 480, and 720 min of 1 nM Heregulin (HRG) stimulation^15^. Dose-response data of phospho-AKT and phospho-ERK upon HRG stimulation at 0, 0.05 and 1 nM were from Neve et al.^15^. Dose-response of phospho-AKT T308 and phospho-ERK T202/Y204 in T47D cells upon insulin stimulation at 0.1, 1, 5, 10 and 20 nM were generated in this study (Fig. S1A). Quantified values display single data points. **c** Generation of BYL719-resistant T47D pools (RPs). **d** 2D growth assays of parental and RPs under steady state (left) or on 1 μM BYL719 (right). Inset: crystal violet of parental and RP1 at day 6 of culture. Data are represented as mean ± SD. One-way ANOVA. ****p < 0.0001 (*n* = 3 replicates of culture). **e** Model prediction of signaling response to BYL719 in parental T47D cells. Blue and red lines indicate parental and resistant model, respectively. Solid lines denote mean values, error bars denote standard error (*n* = 77 best-fitted parameter sets), *p < 0.05. **f** Experimental validation using data in (**g**) and Supplementary Fig. 2A, B. Data points are presented as mean ± SE *p < 0.05 (*n* > 3 independent experiments). **g** Parental and RP1 T47D cells were treated with 1 μM BYL719 for the indicated times; phosphorylation of candidate proteins were monitored by WB.

Cancer complexities undermine efficacy of anti-cancer treatments, but identification of cellular and molecular contexts provides opportunities for more effective therapies. *PIK3CA* encodes the p110α subunit of PI3K and is mutated in ~40% of luminal, estrogen receptor positive (ER+) breast cancer^1^. PI3K phosphorylates PIP2 and generates PIP3, which activates several effector targets to sustain cellular growth. The 3-phosphoinositide-dependent protein kinase 1, PDK1 and AKT are critical mediators of the PI3K signaling output. PDK1 phosphorylates AKT on T308, and a second phosphorylation event catalyzed by the mTOR complex 2 (mTORC2) on AKT S473, leads to full AKT activation and promotion of cell growth and proliferation (Fig. 1a)^2^.

Numerous drug agents have been developed to inhibit PI3K pathway components for cancer treatment^3^. The PI3Kα inhibitor alpelisib, i.e., BYL719, has been approved for the treatment of *PIK3CA*-mutant, ER+ metastatic breast cancer and is in clinical testing for additional malignancies^4^. However, PI3Kα inhibitors in monotherapy fail to induce stable tumor remission due to a number of factors, including dynamic reactivation of growth-promoting signaling nodes which cause treatment failure^5^. Acquisition of secondary mutations in compensatory genes also contributes to resistance to PI3Kα inhibition^6^, highlighting the urgent need to identify co-targets that complement single PI3Kα-targeting agents.

To this end, studies have found that several markers of resistance to PI3Kα inhibition are linked to reactivation of members of the PI3K pathway. These include mTORC1^7,8^, p110β^9^, and activation of the PDK1-SGK1 axis^10^. Inhibition of the Retinoblastoma tumor suppressor, Rb, via selection of high levels of cyclin D1, was also reported to cause resistance to BYL719^11^, indicating that cell cycle regulators play a critical role in moderating cellular responses to this drug.

The discovery of various mechanisms of resistance to PI3Kα inhibition in recent years has led to the development of combination therapies that rescue the sensitivity to BYL719 in pre-clinical models of breast cancer^5^. However, the wealth of combinatorial treatments for BYL719-resistant breast cancer also raises the challenge of how to best prioritize the application of these new targeted therapies to improve patients’ outcomes. Moreover, whether additional PI3K pathway-independent mechanisms contribute to the resistance to PI3K-based therapies in breast cancer has not been fully explored.

By integrating previously identified mechanisms causing resistance to BYL719, we have constructed quantitative and predictive mathematical models representing the PI3K signaling network under a BYL719-sensitive versus BYL719-resistant cell state. Using in silico modeling, we tested how targeted inhibition of each member of the PI3K network synergized with PI3Kα inhibition, and the effect this combination would have on cell proliferation. We found that PDK1 was the top predicted co-target with PI3Kα suppressing activation of critical biomarkers of resistance and enhancing response to BYL719 in parental and BYL719-resistant T47D cells. Through phosphoproteomics we also found that control of DNA damage response pathways by the cyclin-dependent kinase (CDK) inhibitor p21 hides an unexpected vulnerability of BYL719-resistant cells. Consistently, targeting the Checkpoint kinase 1, CHK1, specifically caused death of BYL719-resistant T47D cells. Altogether, these studies reveal the contribution of unappreciated markers of resistance to PI3Kα inhibition, and provide an integrated framework that can be exploited for the identification of more tailored treatments for breast cancer patients.

## Results

### Integrated computational models capture signal dynamics of PI3K network

To provide a systems-level understanding of signaling dynamics occurring upon drug perturbations, including PI3Kα inhibition, we developed a mechanistic computational model that captures the complexity of the PI3K signaling network. The model was formulated using ordinary differential equations (ODEs) describing biochemical interactions as a series of ODEs based on established kinetic laws^12^. The model integrates canonical components of the PI3K pathway and contains reported signaling hubs implicated in the acquired resistance to BYL719 (Fig. 1a). These include: p110α and β-isoforms of PI3K and key downstream effectors (AKT/mTOR/S6K1, SGK3/NDRG1, SGK1/FOXO3, P-Rex1/Rac1, c-Myc); parallel signaling cascades (PI3K/AKT/mTOR, Ras/Raf/MEK/ERK, ERα) and prominent receptor tyrosine kinases (RTKs) such as IGFR/IR and ErbB; nodes converging on critical cell cycle checkpoints (CDKs/Rb/Cyclin D/E and p21) (Fig. 1a). Importantly, the PI3K model also encapsulates feedback and feed-forward molecular loops together with reported crosstalk mechanisms: AKT/mTORC1/S6K1/IRS; AKT/FOXO3/ErbB; CyclinD/CDKs/TSC2/mTORC1. Description of model generation, model reactions, ODE equations, model scope and assumptions are included in Supplementary Information.

To provide context specificity to the in silico representation of the PI3K network, we calibrated our model against data obtained from the ER^+^, T47D human breast cancer cell line harboring the *PIK3CA* H1047R mutation, and sensitive to BYL719^13^. Model calibration involves estimation of unknown model parameters to minimize mismatch between experimental data and simulated outputs^12,14^. To perform this, we employed a combination of new kinetic and dose-response data together with published reports monitoring phosphorylation and total levels of multiple nodes of the PI3K network under distinct stimuli, IGF-1/Insulin and HRG (Fig. 1b and Supplementary Fig. 1A)^15,16^. Parameter estimation was implemented using a genetic algorithm-based optimization procedure coded in MATLAB (Supplementary Information). Given the large size of our model, we assumed that multiple parameters would likely fit the experimental data equally well, a phenomenon commonly known as “model unidentifiability”^16^. To mitigate potential biases of using a single best-fitted parameter set, and overcome model unidentifiability, we employed an ensemble approach whereby we repeated the parameter estimation process with different starting points of parameter values, and obtained multiple parameters sets (*n* = 77) that fitted the data with similar high quality. These parameters sets were collectively used for subsequent simulations (Supplementary Fig. 1B and Supplementary Dataset 1) and demonstrated a faithful replication of experimental data (Fig. 1b). We refer to this calibrated model the ‘parental PI3K model’ as it describes the state of the BYL719-sensitive, parental T47D cell line.

To independently validate the parental PI3K model, we generated new predictions and compared these to new experimental data. We simulated dynamic activation of multiple network proteins in T47D cells in response to growth-media stimulation over 24 h, with or without BYL719, and performed corresponding experiments for validation (Supplementary Fig. 1C, D). Simulations correctly predicted that inhibition of key signaling markers such as ERK, AKT, S6 and Rb would occur in T47D cells in response to BYL719 (Supplementary Fig. 1D). These iterative computational-experimental analyses allowed us to build a predictive model tailored to study signaling dynamics in T47D cells.

To generate a computational model that recapitulates a cell-state associated with resistance to PI3Kα inhibition, we established pools of BYL719-resistant T47D cells, i.e., Resistant Pools (RP) 1, RP2 and RP3 (Fig. 1c). We performed 2D and 3D growth assays and found that BYL719 had a potent cytostatic effect on parental cells while BYL719-RPs kept proliferating even under high BYL719 concentration, confirming their resistant behavior (Fig. 1c, d and Supplementary Fig. 1E, F). We then profiled phosphorylation and total levels of key components of the PI3K network in RPs and T47D parental cells under standard growing condition, and used these data to adjust the parental model and generate the ‘resistant PI3K model’ (Supplementary Fig. 2A, B and Supplementary Information). We further validated both models by generating predictions of temporal changes in phosphorylation levels of important molecular hubs (AKT, ERK and S6) upon BYL719 treatment. Model simulations showed rapid inactivation of these signaling molecules in parental and RPs upon drug treatment (Fig. 1e) but also predicted that levels of phospho-S6 would remain higher in RPs than parental T47D cells. Time-course characterization of phospho-AKT, phospho-ERK and phospho-S6 in response to BYL719 confirmed model predictions (Fig. 1f, g and Supplementary Fig. 2C, D) and was consistent with previous reports^7,8^.

Collectively, we conclude that our parental and resistant-PI3K models faithfully recapitulate signaling dynamics observed in BYL719-sensitive and resistant T47D cells and define new quantitative tools to explore signaling dynamics in defined molecular settings.

### Computational modeling prioritizes synergism between drug combinations

To prioritize efficacy of combinatorial therapies for BYL719-resistant breast cancer, we exploited our computational models and simulated the effect of various pair-wise drug combinations directed at PI3Kα and other components of the PI3K network. We interrogated 24 network nodes and simulated the consequences of inhibiting each of them along with PI3Kα. To assess the efficacy of each drug combination, we used cyclin D1, Rb, and S6 as molecular readouts given their established role in promoting cell growth and resistance to BYL719^7,8,11^. Moreover, higher cyclin D1 levels and phospho-S6, and reduced Rb activity were found in our RPs compared to parental T47D cells (Supplementary Fig. 3A).

**Fig. 2:**
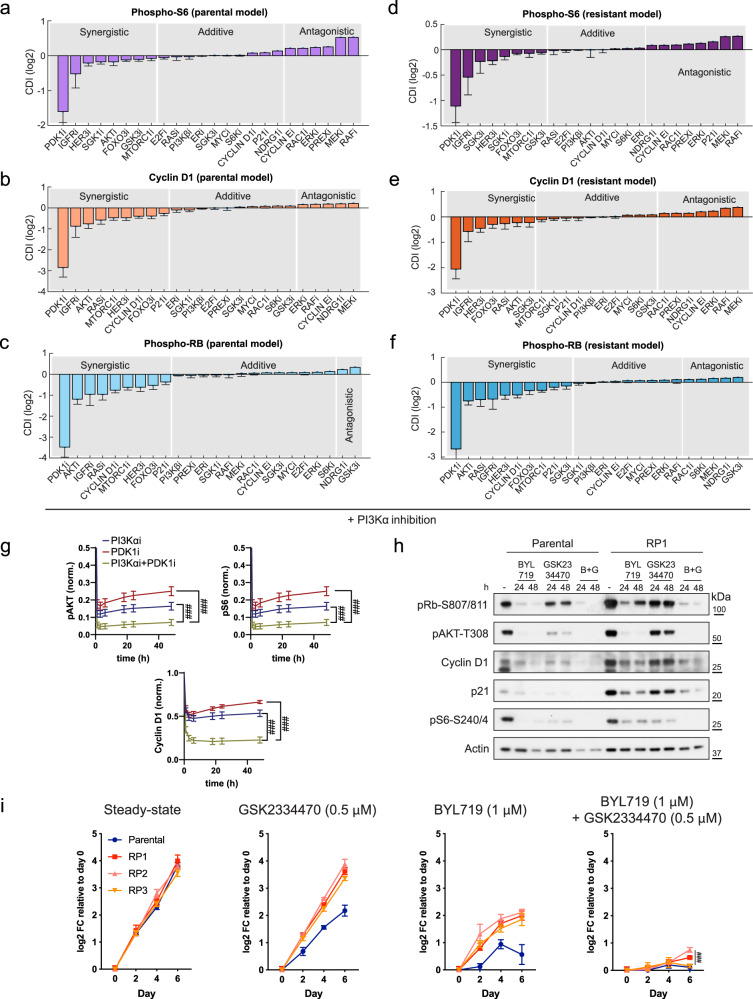
Computational model predicts synergistic drug combinations. **a**–**f** Model prediction of drug synergism (synergy index, log2 scale) was generated using coefficient of drug interaction (CDI) index based on phospho-S6, cyclin D1 levels, or phospho-Rb. Bars indicate mean values ± standard error (see Supplementary Dataset 2). **g** Simulation of phospho-AKT, phospho-S6, and cyclin D1 levels in response to BYL719 and GSK2334470. Solid lines denote mean values, error bar denotes standard error (*n* = 77 best-fitted parameter sets), ^###^*p* < 0.001. **h** WB of T47D parental and resistant cells (RP1) treated with BYL719 (1 μM) or GSK2334470 (0.5 μM), either alone or in combination, for 24 and 48 h. **i** Growth curves of T47D cells treated with BYL719 (1 μM) and GSK2334470 (0.5 μM), either alone or in combination, for 6 days. Drugs were added to the medium every 2 days. Data points are presented as mean ± SD. Student’s *t* test comparison between BYL719/GSK2334470 co-treatment and BYL719 alone of the RP1, RP2 and RP3 on day 6 culture, ^###^*p* < 0.001 (*n* = 3 replicates of culture).

In silico simulations compared the effect of inhibiting either PI3Kα alone, one single PI3K network target, or PI3Kα plus a network target, and assessed the synergism of each pair-wise drug combination on cyclin D1 levels and phosphorylated Rb and S6 (Fig. 2a–f and Supplementary Fig. 3C, D). Possible synergistic effects (or lack thereof) were quantified using the coefficient of drug interaction (CDI) index where CDI < 1, =1, >1 indicate a synergistic, additive, or antagonistic effect, respectively^17^. Accordingly, CDI values allowed us to rank simulated drug combinations and prioritize the most synergistic pairs for experimental validation. We found strong consistency between predicted drug combinations ranking across the molecular readouts indicating robustness in model predictions (Supplementary Fig. 3B).

As previously indicated, our model simulations consistently found that co-targeting IGF1R^8^, members of the SGK family of kinases^10^, mTORC1^7^ with BYL719, would reduce cyclin D1 levels, promote Rb reactivation, and also inhibit S6 phosphorylation in parental cells (Fig. 2a–c and Supplementary Fig. 3C) and RPs (Fig. 2d–f and Supplementary Fig. 3D), thus affecting the status of biomarkers of resistance to BYL719. Notably, the top predicted synergistic co-target with PI3Kα, for both parental and resistant model, was PDK1 (Fig. 2a–f). Time-course simulations showed that while single PI3Kα or PDK1 inhibition blocked major signaling markers (phospho-AKT, phospho-S6, and cyclin D1) to some extent (Fig. 2g), combined PI3Kα plus PDK1 inhibition markedly and durably inhibited these signals over 48 h of drug treatment (Fig. 2g). To validate this, we treated parental and RP cells with BYL719 and the PDK1 inhibitor GSK2334470, either in single or in combination, and measured the dynamic responses of phospho-Rb, Cyclin D1, phospho-AKT and phospho-S6 over 48 h (Fig. 2h). We found that BYL719 plus GSK2334470 suppressed these growth-promoting markers more potently than single-drug treatments, at 24 and 48 h (Fig. 2h and Supplementary Fig. 3E) thus demonstrating durable synergism. Importantly, BYL719 plus GSK2334470 significantly blocked growth of parental and BYL719-resistant cells (Fig. 2i) confirming the superior anti-proliferative effect.

Finally, to determine the suitability of PDK1 and PI3Kα as therapeutic co-targets, we explored genetic dependencies using the Cancer Dependency Map (DEPMAP) database^17^. We found that compared to cells with wild-type *PIK3CA* (*n* = 13)*, PIK3CA*-mutant breast cancer cell lines (*n* = 7) displayed increased dependency on PDK1 and were more susceptible to PDK1 knock-out for their proliferative capacity (Supplementary Fig. 3F).

Together, these integrated computational and experimental analyses support the notion whereby co-targeting PI3Kα with PDK1 defines an optimal strategy to fully inhibit PI3K pathway in *PIK3CA*-mutant cells resistant to BYL719.

### Selection of high p21 levels promotes resistance to PI3Kα inhibition

**Fig. 3:**
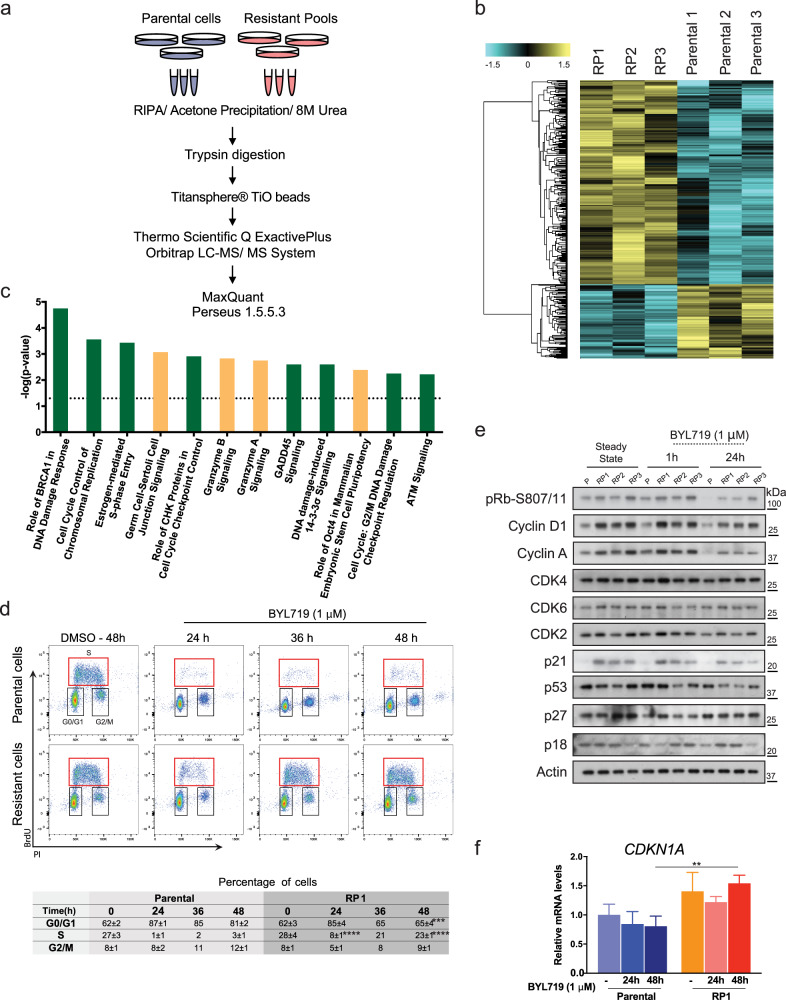
Phosphoproteomics identifies cell cycle checkpoints and DNA damage response pathways enriched in BYL719-resistant T47D cells. **a** Phosphoproteomics work-flow: total protein lysates of T47D parental cells and RPs were processed and phosphopeptides enriched through TiO_2_ beads. Phosphopeptides were quantified using data-dependent acquisition (DDA)-MS analysis. Raw data were imported into MaxQuant to generate relative quantities; statistical analysis and data clustering were performed using Perseus v1.5.5.3, and pathway analysis using Ingenuity Pathway Analysis, IPA. **b** Heatmap of significantly changed phosphosites (425) between RP and parental cells (Student’s *t* test; *p* < 0.05, *n* = 3 biologically independent experiments). The phosphosite intensities were *Z*-score normalized followed by unsupervised hierarchical clustering analysis. Phosphosites in yellow are intensities higher than the mean and in cyan are intensities lower than the mean of the respective phosphosites across all samples. **c** Top enriched canonical pathways identified in RPs compared to parental T47D cells. Green bars indicate cell cycle and DNA damage pathways. **d** Cell cycle profiles of T47D cells based on BrdU incorporation and propidium iodide (PI) staining using flow cytometry (top). T47D cells were treated with vehicle, DMSO (0.1% v/v) or BYL719 (1 μM) for the indicated durations. Bottom, table summarizing percentages of cells (mean ± SD) in different phases of cell cycle, *n* = 3 independent experiments. ***p < 0.001 and ****p < 0.0001. **e** WB of T47D cells left untreated or treated with BYL719 (1 μM) for 1 and 24 h, and probed with the indicated antibodies. **f** qPCR quantifying *CDKN1A* mRNA levels in T47D cells left under growing condition, or treated with 1 μM BYL719 for 24 or 48 h. Data are presented as mean ± SD, **p < 0.01 (*n* = 3 replicates of culture).

To explore whether additional signaling axes outside the PI3K network contribute to BYL719 resistance, we conducted quantitative mass spectrometry (MS)-based phosphoproteomics. Parental and RPs cells were processed according to the label-free quantitative MS method (Fig. 3a) and phospho-peptides quantified (Fig. 3b and Supplementary Fig. 4A). Enrichment of phospho-Ser/Thr and Tyr peptides allowed us to identify 8495 phospho-peptides and 6065 total peptides (Fig. 3b). Ingenuity Pathway Analysis (IPA)^18^ revealed that control of cell cycle checkpoints and response to DNA damage pathways were both highly enriched in RPs relative to parental, sensitive T47D cells (Fig. 3c). Consistently, cell cycle analysis by flow cytometry confirmed that BYL719 had a potent cytostatic effect on parental cells, which arrested in G0-G1 and G2-M phases, whereas RPs kept cycling and dividing irrespective of the inhibitor (Fig. 3d).

Next, we monitored activation status and expression level of key members of cell cycle checkpoints by Western Blotting (WB) (Fig. 3e). In addition to confirming high levels of cyclin D1 and phospho-Rb, we noticed that RPs showed an unexpected increase in the levels of the CDK inhibitor p21 compared to parental cells (Fig. 3e). These changes were accompanied by increases at the mRNA level, indicating a likely transcriptional regulation (Fig. 3f). Interestingly, this condition was unique to p21 as other CDK inhibitors (p27, p18 and p53) showed no alterations across cell lines (Fig. 3e). However, since T47D cells express a mutant p53 (p53L194F), to ascertain its biological role in this context, we knock-out p53 in RP2s, generating RP2-p53KO cells, and found that upon complete p53 depletion, p21 levels remained readily detectable (Supplementary Fig. 4B). Furthermore, 2D growth assays showed that the loss of p53 did not impair the proliferation of RP2-p53KO cells in comparison to RP2-Scr (Supplementary Fig. 4C), indicating that the p53 L194F mutation does not exert growth-promoting effects and that p21 expression in BYL719-resistant cells is driven by p53-independent mechanisms.

**Fig. 4:**
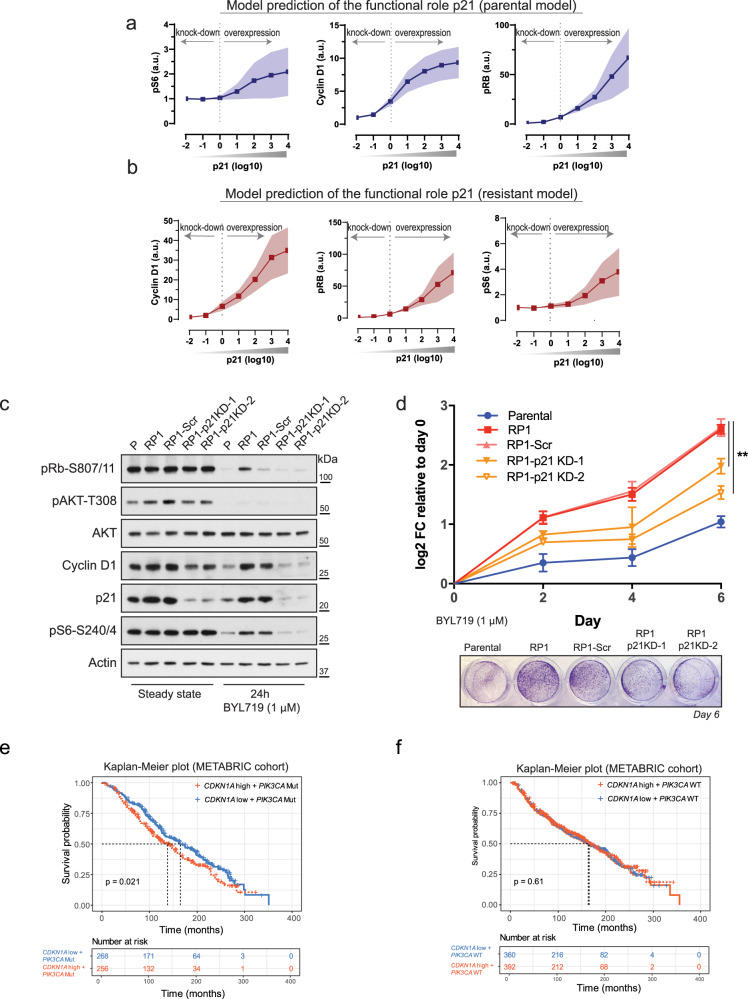
p21 knock-down rescues sensitivity of RPs to BYL719. **a**, **b** Model predictions assessing inter-dependency between p21 levels and biomarkers of resistance to BYL719. Increases in p21 level correlate with higher cyclin D1 levels, increased Rb and S6 phosphorylation, in parental and resistant models. Solid lines indicate mean values, shaded areas indicate standard errors (*n* = 77 best-fitted parameter sets). Simulation was carried out in 1 μM BYL719 treatment condition. **c** WB of T47D parental cells or with targeted p21 knock-down (KD). CRISPR/Cas9 was used to transduce RPs with scramble (Scr) (RP1-Scr) or two independent guide-RNAs directed at *CDKN1A* (RP1-p21KD-1, and RP1-p21KD-2). Cells were left either untreated or treated with 1 μM BYL719 for 24 h. See also Supplementary Fig. S5A. **d** *CDKN1A* knock-down re-sensitizes T47D RP1 cells to BYL719. The T47D cell series was treated with 1 μM BYL719, refreshed every 2 days for 6 days. Crystal violet at day 6 of treatment, bottom. Data are presented as mean ± SD. One-way ANOVA, **p < 0.01 (*n* = 3 replicates of culture). See also Supplementary Fig. 5B. **e** Breast cancer patients (METABRIC) with alterations in *PIK3CA* (Mutations, Mut) and expressing high *CDKN1A* levels associate with poorer patients’ outcomes compared to patients with *PIK3CA* mutations but low *CDKN1A* (*n* = 524 patients). **f** Probability of overall survival of breast cancer patients harboring wildtype *PIK3CA* and high *CDKN1A* levels (*n* = 752 patients).

Next, to assess whether p21 was able to regulate the proliferative capacity of RPs, we utilized our computational models and simulated the effect of varying p21 levels on key markers of cell cycle progression and proliferation. Using the parental PI3K model, we predicted that graded increases in p21 levels would upregulate cyclin D1, inhibit Rb, and increase phospho-S6 (Fig. 4a). Moreover, the resistant PI3K model estimated that diminishing p21 levels in RPs would reduce cyclin D1 and phospho-S6 levels, and reactivate Rb, indicating the presence of a crosstalk between these molecules (Fig. 4b). Interestingly, others have previously shown that p21 can positively regulate formation of the cyclin D1-CDK4/CDK6 complex^19^.

To confirm these predictions and validate a role for p21 in promoting survival of RPs, we targeted *CDKN1A* (encoding p21) with two independent guide RNAs, and generated RP-p21 knock-down (KD) T47D lines. We found that reduced p21 levels decreased cyclin D1 levels (Fig. 4c and Supplementary Fig. 5A) and significantly impaired the growth capacity of all RPs (Fig. 4d and Supplementary Fig. 5B). Our data also showed that p21KD suppressed phosphorylation of Rb and S6 (Fig. 4c and Supplementary Fig. 5A) and hence abated critical pro-survival signals. Moreover, considering that the functional output of p21 can also be influenced by its localization^20^, we next quantified the cellular distribution of p21 in the T47D cell series by immunofluorescence (IF). In cells maintained in growing conditions, we observed a nearly equal distribution of p21 between the nucleus and cytoplasm. However, upon BYL719 treatment, there was a marked increase in p21 nuclear localization across all cell types (Supplementary Fig. 5C). Notably, p21 nuclear distribution was significantly more pronounced in RPs than in T47D cells (Supplementary Fig. 5C). This suggests that not only resistant cells exhibit higher levels of p21, but they also display a more pronounced nuclear accumulation of p21 compared to their parental counterparts.

Finally, we investigated the status of p21 in breast cancer patients using public datasets (METABRIC and TCGA). We confirmed that ~4% of breast cancer patients displayed amplification/ increased expression of *CDKN1A* compared to average levels across all samples (Supplementary Fig. 5D)^20^. We then focused on breast cancer samples with *PIK3CA* mutations and observed that *PIK3CA*-mutant patients with high *CDKN1A* expression were associated with poorer overall patients survival compared to patients with mutant PI3K but lower *CDKN1A* levels (Fig. 4e). Notably, this correlation was not found in *PIK3CA* wildtype patients (Fig. 4f). We also studied the association between cyclin D1 and *PIK3CA* mutations given the established oncogenic role of cyclin D1 in breast cancer, and its deregulation in our experimental system. Surprisingly, high *CCND1* levels did not predict outcomes in breast cancer patients, irrespective of the *PIK3CA* status (Supplementary Fig. 5E, F). Thus, these findings suggest a unique cooperation between PI3K and p21 in breast cancer malignancy.

To further corroborate these findings, we next generated T47D cells resistant to the pan-PI3K inhibitor BKM-120. We found that also in response to this alterative targeted therapy, the resulting resistant pools displayed increased p21 levels compared to control cells (Supplementary Fig. 5G). This indicates that selection of high-p21 levels plays a protective, pro-survival role in the face of diverse PI3K-based therapeutic challenges.

### Evasion of cellular senescence overcomes the anti-proliferative effect of BYL719

**Fig. 5:**
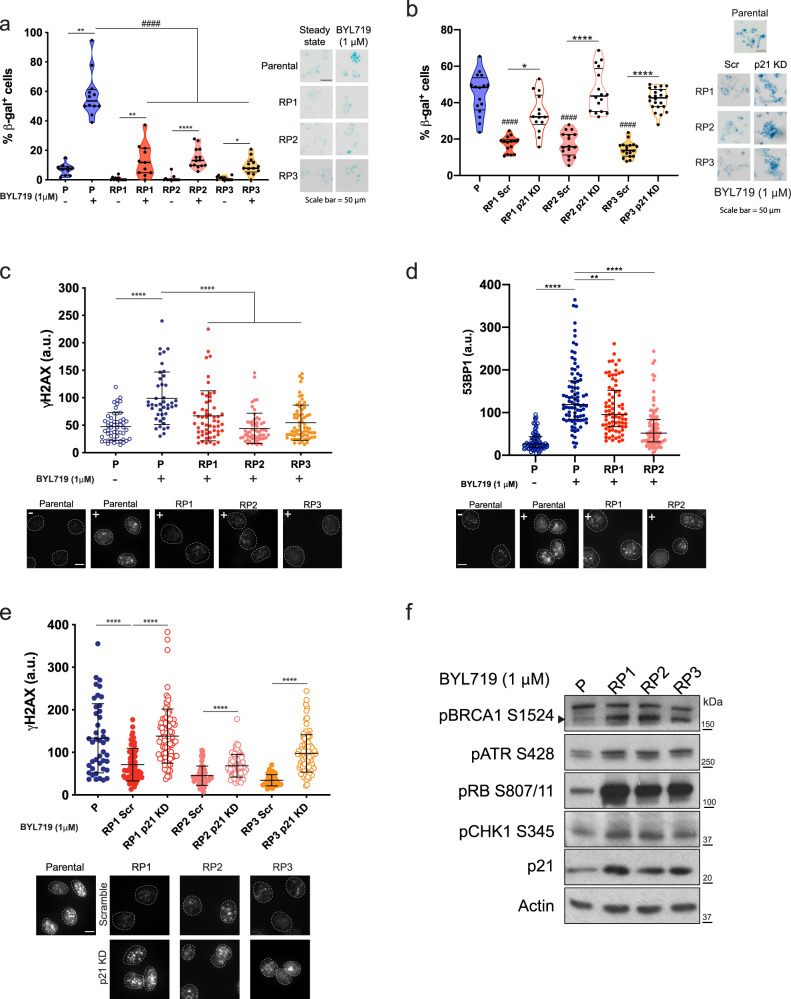
Resistance to BYL719 overcomes DNA-damage induced cellular senescence. **a** β-gal senescence assay of parental and RP cells left untreated or treated with 1 μM BYL719 for 48 h. Percentages of β-gal positive cells were calculated by dividing the number of β-gal (blue) cells over the total number of nuclei (DAPI). Violin plots showing median plus 1st and 3^rd^ quartiles. Kruskal–Wallis analysis, *p < 0.05, **p < 0.01, ****p < 0.0001; ^####^*p* < 0.0001 for comparison between cells in BYL719. Right, β-gal stains (bright-field) of parental and RP cells treated as indicated. Scale bar = 50 μm (*n* = 10 fields of view). **b** Percentage of senescence in RPs with targeted p21 KD, quantified as in (**a**). Right, β-gal staining of T47D cells treated as indicated. Violin plot showing median plus 1st and 3rd quantiles. Kruskal–Wallis analysis, *p < 0.05, ****p < 0.0001; ^####^*p* < 0.0001 for comparison between RPs and Parental cells (*n* > 10 fields of view). **c** γH2AX fluorescence intensity was quantified in parental T47D cells and RPs upon 1 μM BYL719 treatment for 48 h; each data point represents one nucleus. Mean ± SD is shown, one-way ANOVA, ****p < 0.0001 (*n* > 40 nuclei per group). Bottom, representative γH2AX images of parental and RPs in 1 μM BYL719 for 48 h. Scale bar = 5 μm. **d** 53BP1 fluorescence intensity was quantified in parental T47D cells and RPs upon 1 μM BYL719 treatment for 48 h; each data point represents one nucleus. Mean ± SD is shown, one-way ANOVA, ****p < 0.0001. Bottom, representative 53BP1 images of parental and RPs in 1 μM BYL719 for 48 h. Scale bar = 5 μm. **e** DNA damage quantification of T47D cells with targeted p21 KD and treated with 1 μM BYL719 for 48 h. γH2AX fluorescence intensity was quantified as in (**d**). Mean ± SD is shown, one-way ANOVA, ****p < 0.0001 (*n* > 40 nuclei per group). Representative images of γH2AX in parental and RPs. Scale bar =5 μm. **f** WB analysis of DNA damage repair activators in T47D parental and RP cells treated with 1 μM BYL719 for 48 h.

The cytostatic effect induced by BYL719 prompted us to test whether this cellular state was associated with therapy-induced senescence (TIS). To test this, we scored percentages of cells positive to the senescent marker β-galactosidase (β-gal) in parental, RPs and RPs-p21KD T47D cells upon BYL719. We found that parental cells showed low levels of senescence under standard growing conditions (~7%) (Fig. 5a), and that addition of BYL719 for 48 h significantly increased the β-gal positivity (~55%) (Fig. 5a). RPs also showed low senescence in standard conditions (~3%), but the addition of BYL719 only mildly increased the percentage of β-gal cells (~13%), which remained significantly lower than that observed in parental cells (Fig. 5a). Next, we quantified the senescence in RP-p21KD pools and found that p21 knock-down in RPs restored percentages of β-gal positivity to similar levels observed in parental cells (~40%) (Fig. 5b). These data collectively indicate that BYL719 treatments induce a potent senescence response in parental T47D cells and that selection of high-p21 contributes to bypassing this cytostatic condition. We also tested whether alterations in p16, another critical regulator of cellular senescence occurred in these cells and found that p16 levels remained unchanged in parental and RPs even when these were treated with BYL719 (Supplementary Fig. 6A). This demonstrates that p21 plays a unique role in overcoming the BYL719-induced senescence response.

We next asked whether PDK1 inhibition, like PI3Kα inhibition, causes TIS. We found that treatment with the PDK1 inhibitor GSK2334470 resulted in a threefold increase in senescence in T47D parental cells compared to DMSO-treated cells (Supplementary Fig. 6B). However, in RPs, GSK2334470 only caused minimal β-gal staining (Supplementary Fig. 6B). This indicates that similar to PI3K inhibition, PDK1 inhibition triggers senescence in sensitive cells, albeit to a lesser extent than that observed with BYL719. Moreover, in 2D growth assays (Fig. 2i) we observed that BYL719-RPs proliferated more rapidly than parental T47D cells even when treated with GSK2334470, indicating that acquisition of resistance to PI3K inhibition also confers resistance to single PDK1 inhibition.

### Genomic instability is a vulnerability of BYL719-resistant breast cancer cells

Senescence defines a quiescent cell state that can be induced by DNA damage. To explore whether the senescence observed in BYL719-treated T47D cells was associated with damage to DNA, we immuno-stained the T47D cell series with the DNA damage marker γ-H2AX and 53BP1. We found that 48 h of BYL719 treatment doubled the γ-H2AX positivity in parental cells, reaching levels significantly higher than those observed in RPs (Fig. 5c). Similarly, T47D cells showed diffuse 53BP1 nuclear staining in DMSO which became localized in intense nuclear foci upon BYL719 treatment; similar to γ-H2AX, the intensity of 53BP1 nuclear foci was higher in parental cells than RPs (Fig. 5d).

Importantly, p21KD rescued the γ-H2AX intensity in RPs, i.e., RP-p21KD cells, compared to control samples (Fig. 5e). This led us to conclude that prolonged PI3K inhibition causes DNA damage-induced senescence and that selection of high-p21 levels contributes to repair the damaged DNA and promotes exit from this arrested cell state.

**Fig. 6:**
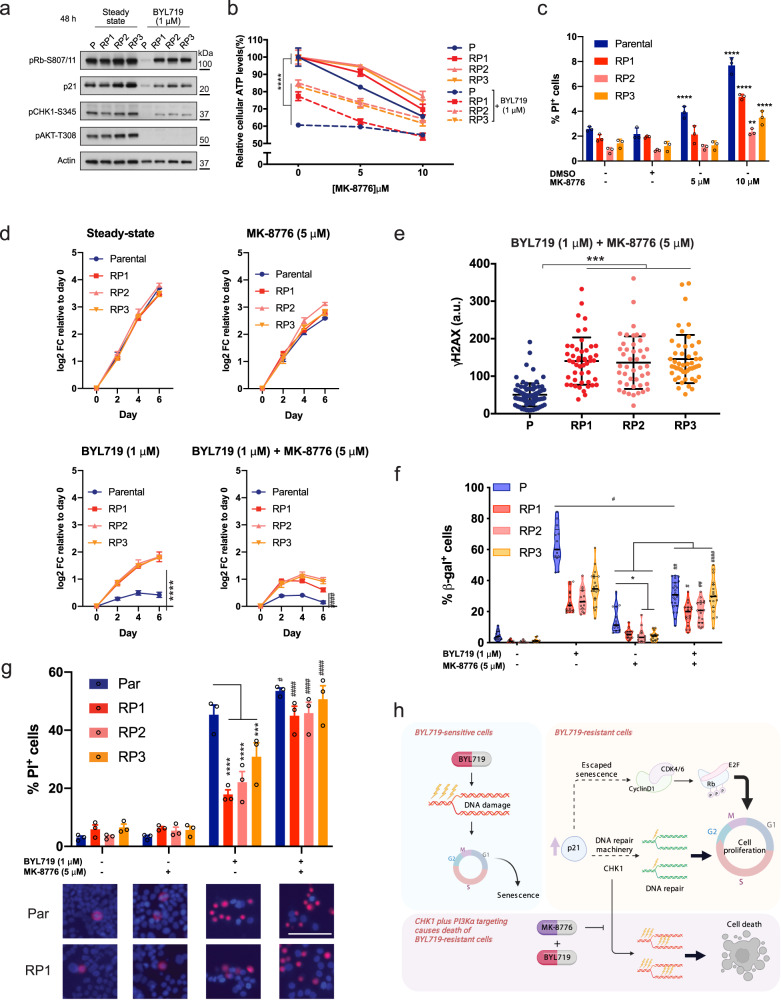
CHK1 inhibition sensitizes BYL719-resistant T47D cells to death. **a** WB of parental and RPs treated with 1 μM BYL719 for 24 h and probed with the indicated antibodies. **b** T47D parental cells and RPs were treated with 1 μM BYL719 alone or in combination with either 5 or 10 μM of the CHK1 inhibitor MK-8776 for 3 days. Cell viability was quantified using CellTiter-glo, Data presented as mean ± SD, one-way ANOVA, ****p < 0.0001 (*n* = 3). **c** T47D parental cells and BYL719-RPs were treated with the indicated doses of MK-8776 for 4 days and percentage of dead cells quantified through PI staining. Data presented as mean ± SD, one-way ANOVA comparison between DMSO and MK-8776 treatments of the same cell line, **p < 0.01. ****p < 0.0001 (*n* = 3). **d** Growth curve of T47D cells treated with BYL719 (1 μM) or MK-8776 (5 μM) alone or in combination for 6 days. Drugs were refreshed every 2 days. Data points represent mean ± SD, one-way ANOVA, ****p < 0.0001, Student’s *t* test, ^###^*p* < 0.001 comparing response to BYL719 alone or in combination with MK-8776 between RPs and parental cells (*n* = 3). **e** DNA damage quantification in parental and RPs treated with 1 μM BYL719 and 5 μM MK-8776 for 48 h. γH2AX fluorescence intensity was quantified and each data point represents one nucleus. Mean ± SD is shown, one-way ANOVA, ***p < 0.001 (*n* > 40 nuclei per group). **f** β-gal assay of parental and RPs treated with BYL719 (1 μM) or MK-8776 (5 μM) alone or in combination for 4 days. Percentage of positive cells was calculated as the number of β-gal positive cells over total number of nuclei. Violin plot showing median plus 1st and 3rd quantiles. Kruskal–Wallis analysis, *p < 0.05; ^#^*p* < 0.05, ^##^*p* < 0.01, ^####^*p* < 0.0001 for comparison between treatments of the same cells (*n* > 10 fields of view from each triplicate wells). **g** Cell death assay of T47D parental cells and RPs treated with BYL719 (1 μM) or MK-8776 (5 μM) alone or in combination for 6 days. Number of dead cells were quantified through PI staining and normalized over total cell number quantified by Hoechst 33342 dye. Data are presented as mean ± SEM, one-way ANOVA, ***p < 0.001, ****p < 0.0001; ^#^*p* < 0.05, ^####^*p* < 0.0001 for comparison with BYL719 treated cells (*n* = 3 replicates of culture). Inserts show PI-stained (red) T47D parental cells and RP1 cells in different conditions. Nuclei counterstained with Hoechst33342 (blue). Scale bar = 100 μm. **h** BYL719 treatments cause DNA damage and senescence in T47D cells. Selection of high-p21 levels promotes repair of damaged DNA and evasion of drug-induced cellular senescence. p21 can also promote formation of the cyclin D1-CDK4/CDK6 complex, further supporting cell cycle progression^19,116^. In combination with BYL719, the CHK1 inhibitor MK-8776 leads to excessive DNA damage and death of BYL719-resistant cells.

Given the increased DNA damage caused by BYL719 in T47D cells, we next wondered whether targeting DNA damage response pathways could provide a valuable approach to complement PI3Kα inhibitors in the treatment of resistant cells. To this end, we focused on essential regulators of the G2-to-M cell cycle checkpoints whose blockade induces death of p53-mutant cells, such as the T47D cells^21^. First, we found that RPs showed higher phosphorylation levels of CHK1-Ser345, ATR-S428 and BRCA-S1524, confirming that these cells have heightened activation of DNA damage repair and replication checkpoints (Figs. 5f and 6a). Second, we tested the cell viability of T47D cells upon increasing concentrations of the CHK1 inhibitor MK-8776 (Fig. 6b). We found that 3 days of MK-8776 treatment mildly but significantly limited survival of parental cells and RPs, and caused a small percentage of cell death (Fig. 6c). However, in combination with BYL719, CHK1 inhibition effectively constrained cell growth in parental cells (Fig. 6b) and also rescued the sensitivity of RPs to BYL719 (Fig. 6d). Importantly, this effect was associated with increased DNA damage (Fig. 6e), which not only partially rescued the senescence response in RPs (Fig. 6f), but significantly enhanced their cell death compared to single-drug treatments (Fig. 6g).

These data indicate that blocking CHK1 in combination with BYL79 provides an effective therapeutic approach to specifically target and kill BYL719 resistant cells (Fig. 6h).

### Extending the applicability of experimental and in silico models

**Fig. 7:**
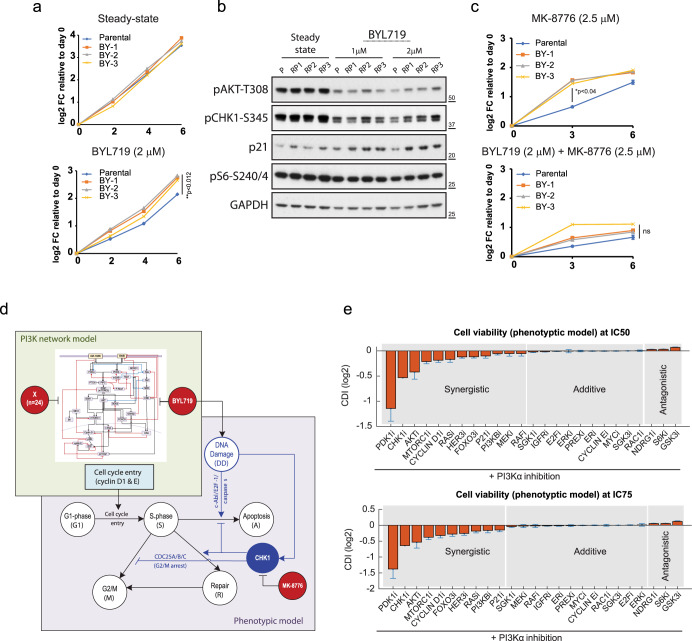
High-p21 and increased CHK1 activation in Saos2 BYL719-resistant versus parental cells. **a** Cell growth assays shown as log2 fold-change (FC) of Saos2 parental and resistant pools (BYs) in either DMSO (Steady-state) or 2 μM BYL719. **b** WB showing increased levels of p21 and pCHK1-S345 in resistant compared to parental Saos2 cells treated with 1 and 2 μM BYL719 for 24 h. **c** Growth curves showing that BYL719 and MK-8776 better suppress growth of Saos2 BYs than single treatments. **d** The phenotypic model: a phenomenological model containing major cell cycle phases (encompassing G1, S, G2/M phases) and key biological outcomes, such as DNA Repair (R) and Cell Apoptosis (A). See also Supplementary Table 4 and Supplementary Dataset 3 for rate equation and kinetic parameters. BYL: PI3Kα inhibitor, BYL719. MK: CHK1 inhibitor, MK-8776. Arrow: activation, bar-headed arrow: inhibition process. **e** Model simulations of drug synergism for 25 drug pairings co-targeting PI3K. Error bars indicate mean values ± standard error (*n* = 77). Single drugs are combined using either their IC50 (top panel) or an IC75 (bottom panel) concentration, displaying consistent results.

To broaden the scope of our findings, we tested the response of the osteosarcoma Saos2 cell line to BYL719, as these cells demonstrated resistance to anti-cancer treatments following induced p21 expression^22^. To this end, we chronically exposed three independent plates of Saos2 cells to increasing concentrations of BYL719 (starting at IC50: 2 μM), or DMSO (similar v/v) as control. We generated 3 BYL719-resistant cell lines (BY-1, BY-2, and BY-3) and confirmed that they proliferated more rapidly than control cells in the presence of BYL719 (Fig. 7a). Importantly, we detected elevated levels of p21 and CHK1 phosphorylation in BYL719-resistant cells compared to control samples (Fig. 7b). Consistently, a combination treatment of BYL719 and the CHK1 inhibitor MK-8776 effectively suppressed the growth of both cell types. These data further support a role for high-p21 in drug resistance, and highlight the efficacy of co-targeting PI3K and CHK1 in therapy-resistant cells, especially those with mutations in PI3K and p53 (Fig. 7c).

We next sought to evolve the in silico PI3K network model to generate a phenomenological model that integrates major cell cycle phases (encompassing G1, S, G2/M phases) and key phenotypic outputs (i.e., DNA Repair, and Apoptosis). This *phenotypic model* also incorporates DNA Damage (DD) and CHK1 activity as explicit model components to trigger biological outcomes: DNA repair and cell viability, or apoptosis (Fig. 7d). Using a similar simulation approach as with the original model, we determined the impact of co-inhibiting each node of the evolved phenotypic model (*n* = 25, including CHK1) in conjunction with PI3Kα on cell viability. A detailed description of the construction of the new model along with model assumptions is provided in the 'Construction of the phenotypic model‘ section of the Methods; while detailed rate equations and kinetic parameters are provided as Supplementary Table S4 and Supplementary Dataset 3.

Model simulations revealed that co-inhibition of PI3Kα and CHK1 exhibited a marked synergistic effect on suppressing cell viability, ranked as the second most potent combination among all the evaluated pairs (Fig. 7e). Co-inhibition of PDK1 and PI3K remained the most synergistic combination (see Fig. 2a–f), indicating the essential role of full PI3K pathway inhibition for cancer suppression. Notably, the order of synergistic drug pairings obtained from the phenotypic model closely aligned with the predictions from the original PI3K model, reinforcing the experimental synergy observed with CHK1 and PI3K inhibitors.

## Discussion

We have integrated computational and experimental approaches to tackle an important question in breast cancer treatment: resistance to oncogene-based targeted therapies. We have studied molecular responses of a *PIK3CA*-mutant, ER+ breast cancer cell line to short-term and chronic exposure to BYL719, and defined signaling adaptations within the PI3K network that cause drug resistance. Our model predictions show that PDK1 is the most effective target enhancing the antitumor activity of BYL719 in sensitive and resistant T47D breast cancer cells. Modeling showed that PDK1 plus PI3Kα inhibition display the highest synergistic drug-score, superior also to compounds targeting mTORC1 or cyclin D1 (Fig. 2a–f), which translated into stable inhibition of markers of proliferation and growth suppression of resistant cells (Fig. 2h, i). Thus, consistent with studies emphasizing a role for PDK1 in promoting breast cancer malignancy^23^, our predictions indicate that PDK1 is also a prominent target for the treatment of resistant disease in ER+ breast cancers with *PIK3CA* mutations. Notably, the in vivo efficacy of this drug combination was previously reported in pre-clinical model of breast cancers^10^. Our modeling also supports the notion whereby in a context in which AKT is still inhibited (Fig. 1g) compensatory PDK1 activation in response to BYL719 rescues a pro-growth signal by engaging with the SGK1/3 kinases^10,24^. Consistently, RPs show higher phosphorylation of the SGK1-target NDRG1 than parental T47D cells (Supplementary Fig. 2A). Thus, by building and training new in silico models, we have generated new tools that accurately predict effective combinatorial therapies directed at key components of the PI3K pathway. These tools can guide the prioritization of combinatorial treatments when monotherapies fail.

Several oncogene based-targeted therapies have been approved for breast cancer, however, lack of predictive biomarkers of response remains a clinical hurdle^5^. Rapalogs and inhibitors of CDK4/CDK6 ribociclib and palbociclib can improve response of ER+/HER2− breast cancer to hormone therapies, but how to best identify which patient benefits from one or the other therapy is unknown^25^. Our in silico approach offers the ability to capture context-specific and dynamics of cellular responses to clinically relevant drugs, and provides a new means to analyze personalized treatments for *PIK3CA*-mutant patients. In future, these models can be integrated with additional datasets into unified quantitative frameworks to rationally design and prioritize therapeutic strategies for different types of breast cancers, or malignancies, with PI3K alterations.

Beyond the PI3K network, we have also shown that T47D cells become resistant to BYL719 thanks to the pro-survival effect induced by the selection of high-p21 levels. Better known as the CDK inhibitor downstream to the tumor suppressor p53, p21 has been shown to regulate several biological processes in response to DNA damage, in a p53-independent manner^20^. Upregulation of p21 in response to oncogene induced DNA-damage (e.g., PML-RARα) was reported to promote DNA repair and contributed to stem cell maintenance in the hematopoietic and mammary epithelial compartments^26,27^. By interacting with the proliferating cell nuclear antigen (PCNA), p21 can displace DNA replication enzymes and blocks DNA synthesis in favor of DNA repair^28^. Consistently, we now show that p21 upregulation also occurs in T47D cells and Saos2 cells upon chronic BYL719 treatments and, by promoting repair of damaged DNA, high-p21 favors bypass of therapy-induced senescence (TIS) (Figs. 5 and 7a–c). Our findings provide multiple new insights into the events that ultimately cause resistance to PI3Kα inhibition. First, we found that the BYL719-induced cytostasis is associated with a senescence response. While TIS has been observed in vitro and in vivo^29^, including human studies in response to chemotherapies^30,31^ we add that this also occurs in response to PI3Kα inhibition, and PDK1 inhibition (Fig. 5a and Supplementary Fig. 6B). Second, we find that the BYL719-induced senescence is associated with DNA damage, consistent with reports showing that the pan-PI3K inhibitor BKM120 and BYL719 itself decrease de novo nucleotide synthesis and cause replication stress in breast cancer models^32^. Third, unlike the common knowledge whereby p21 promotes senescence^20^, we find that selection of high-p21 favors evasion of this cellular defense mechanism by promoting repair of damaged DNA. Evasion of cellular senescence has been associated with selection of tumor cells with stem-like properties^33^ and importantly, that p21 upregulation promoted senescence bypass was previously reported in response to chemotherapeutic agents^22^. In Li Fraumeni-derived fibroblasts and Saos2 osteosarcoma cells with p53 loss, forced p21 expression caused an initial senescence response but also favored selection of escaped cells with even higher p21 levels, aggressive phenotypes, and resistant to doxorubicin and cisplatin^22^. More recently, high-p21 levels were shown to promote tumor growth and resistance to HER2-targeted therapies in *HER2* and *PIK3CA* mutant breast cancers^34^. Thus, we conclude that high-p21 levels can be used as a biomarker to identify tumor cells with potential acquired resistance to systemic and targeted therapies inducing DNA damage.

Further, the dependency between high-p21 and repair of DNA damage is underscored by the efficacy with which CHK1 inhibition and BYL719 increase death rates of RPs (Fig. 6f), but show low toxicity on quiescent, parental T47D cells (Fig. 6c). CHK1 is a target of the ATM/ATR kinases and is activated in response to DNA damage and replication stress^35^, and controls replication initiation forks^36^. CHK1 loss can lead to mitotic catastrophe and death in cells with *Tp53* alterations which have lost control of the G1/S checkpoint, and rely on G2/M checkpoint regulators such as CHK1 to safeguard genome stability^21^. By inhibiting CHK1 activity in cells that depend on DNA repair for survival, such as the BYL719-resistant cells, we propose a new synthetic lethal approach based on PI3Kα inhibition. Importantly, the synergism between CHK1 inhibition and inhibition of PI3K pathway components has been observed also in triple-negative breast cancer (TNBC) cell lines with mutations in PI3K, PTEN, plus p53^37^, and extends to high-grade serous ovarian carcinoma^38^. Thus, in a mutant p53 background, high-p21 level sensitizes cells to compounds that target DNA synthesis and DNA repair, and defines a vulnerability of cells treated with DNA damaging agents, including PI3K inhibitors.

Finally, we assessed whether PDK1 inhibition affected p21 levels in parental and RPs and noticed that when combined with BYL719, GSK2334470 decreased p21 levels (Fig. 2h). Interestingly, p21 protein stability is controlled by the mTORC1 substrate 4EBP1, and active mTORC1 leads to higher p21 protein levels^39^. Therefore, by repressing mTORC1 activation, PDK1 inhibition also affects p21 protein stability and defines a strategy to limit p21 upregulation.

Collectively, in this study we have generated new computational tools and identified critical genetic determinants causing resistance to PI3K-based therapies and propose new combination treatments for *PIK3CA*-mutant resistant breast cancers.

## Methods

### Computational modeling

Mathematical models were formulated using ODEs based on synthesis of model components and network interactions. Detailed accounts of model reactions, reaction rates and equations are included in Supplemenatary Tables S1 and 2. Model construction and simulation were implemented in MATLAB in conjunction with the IQM toolbox (https://iqmtools.intiquan.com/). The parental PI3K model was fitted to experimental data and parameter estimation, and was implemented using genetic algorithms as part of the Global Optimization Toolbox in MATLAB. Values of all best-fitted parameter sets are provided in Supplementary Dataset 1.

The resistant PI3K model was derived from the parental model by adjusting initial conditions of model species between parental and RPs, guided by experimental measurement. Details on model generation, scope and assumptions, as well as model fitting are provided in the Supplemental Information.

### Mathematical model description and assumptions

To systematically and quantitatively interrogate the dynamic cellular response to drug treatments and the adapted behaviors of the PI3K signaling network, we constructed a dynamic mathematical model that integrates canonical components of the PI3K pathway and major reported signaling axes implicated in the acquired resistance to PI3K inhibition.

### Upstream receptor tyrosine kinases (RTKs)

The PI3K pathway sits downstream multiple RTKs including the IGF-1R/IR and ErbB families of tyrosine kinase receptors^40,41^, which have been shown to confer resistance to PI3Kα inhibition^42–44^. Thus, IGF-1R/IR and ErbBs were chosen as single model species, each representing their respective RTKs family. In addition, ErbB2 (HER2) and ErbB3 (HER3) are the most abundantly expressed members of their RTKs family in T47D cells^45^, and act as preferred binding partners among the ErbB family receptors, thus defining prototypical molecules in our model system^46^.

Our model incorporates the p110α and β isoforms of PI3K and their key downstream effector signaling axes, such as AKT/mTOR/S6K1/S6, SGK3/NDRG1, SGK1/FOXO3, P-Rex1/Rac1 and c-Myc (Fig. 1a). Their inclusion is based on studies showing that p110β and SGKs confer resistance to isoform selective or pan-PI3K inhibitors^42,47,48^. Our model also includes the Ras/Raf/MEK/ERK MAPK signaling pathway, a major crosstalk pathway that displays reciprocal interplay with the PI3K signaling and frequently confers resistance to inhibitors targeting PI3K/AKT signaling^41,49^. Both IGF-1R/IR and ErbB can promote activation of the p110α/β/PIP3/PDK1-AGC protein kinase family (AKT, SGK1, and SGK3) pathways; and ErbB directly activates the Ras-Raf-MEK-ERK pathway, as shown in Fig. 1a.

### Cell cycle machinery

An important feature of our mechanistic model is the incorporation of the critical cell cycle machinery (CDKs/Rb/Cyclin D/E/E2F and p21), downstream PI3K/AKT/mTOR and the Ras/MAPK pathway. We assumed that mTORC1 controls the translation of c-Myc and cyclin D1 mRNA by activating eukaryotic translation initiation factor 4E (eIF-4E)^50,51^. The transcriptional repressor activity of Rb can be antagonized by sequential phosphorylation events, initiated by cyclin D1-CDK4/6 in early G1 phase, followed by cyclin E-CDK2 in late phase^52^. Hyper-phosphorylated Rb loses its association with chromatin to release its suppressive role on E2Fs in the nucleus^53^. E2Fs induces expression of cyclin E independently and also in collaboration with Myc^54^, as shown in Fig. 1a. The model also includes the positive auto-regulation of E2F^54^. Expression of p21 was assumed to be regulated by ERK^55^. Furthermore, AKT phosphorylates p21 and enhances its protein stability, which promotes cell survival^56^.

### Major feedback loops and crosstalk mechanisms

Signaling dynamics and drug response behaviors are strongly determined by the presence of positive and negative feedback interactions within signaling networks^41,57^. Thus, our model aimed to capture these events through a careful synthesis of the literature. Below we discuss the salient mechanisms included in the model.

#### Rb-mediated feedback to mTORC2/AKT

We assumed that hyper-phosphorylated Rb directly suppresses the kinase activity of mTORC2 toward its substrate AKT. This was because phosphorylated Rb binds Sin1, an integral component of mTORC2, thereby blocking AKT access to mTORC2, and ultimately leading to attenuated AKT activation^58^. This constitutes a negative feedback loop between Rb and mTORC2/AKT.

#### Cyclin D1-CDK4/6 mediated feedback loop

Another feedback loop emanating from the cell cycle module to an upstream pathway is governed by cyclin D1-CDK4/6 complex, which phosphorylates and inhibits TSC2 at Thr-1462^59^. This effectively generates a positive feedback between Cyclin D1-CDK4/6 and mTORC1, as shown in Fig. 1a.

#### S6K-mediated negative feedback loop

There are a number of known negative feedback loops originating from mTORC1 that ultimately limit PI3K/AKT activation, including one mediated by S6K and one by Grb10, both acting through IRS1/2^2^. Although these feedback mechanisms are biochemically distinct, functionally they act in a similar manner, that is to shut down PI3K/AKT activation when it is exceedingly high. Thus, to keep the model simple yet biologically relevant, we only included the S6K-mediated feedback loop^60–62^.

#### GAB-mediated feedback loop

A major role of PI3K is to catalyze PIP2 phosphorylation to PIP2. In the model, we assumed that PIP3 in turn can recruit the adapter proteins GAB1/2, which enhances PI3Kα and β activation through association with the p85 subunit of PI3K^63–65^. Effectively, these interactions create positive feedback loops between PI3K and GAB1/2.

#### P-Rex1-mediated feedback loop

P-Rex1 is a guanine nucleotide exchange factor (RacGEF) of Rac1, which promotes the activity of Rac1. Interestingly, P-Rex1 is activated by PIP3^66–68^. Moreover, p110β is a direct Rac1 target, and Rac1 activates p110β^69^. Thus, these interactions form a positive feedback loop between P-Rex1 and p110β via Rac1 and PIP3, as shown in Fig. 1a. In addition, P-Rex1 catalyzes the activation of Rac1, which sequentially stimulates the kinase activity of PAK1^70^. PAK1 enhances ERK signaling by phosphorylation of Raf-1 (S338) and MEK1 (S298)^71–73^. For simplicity, we assumed that Rac1 directly phosphorylates MEK.

#### ERK-mediated feedback loops

Multiple negative feedback mechanisms are induced by ERK towards upstream signaling components, including Raf, Ras and RTKs^74^. In our model, we included two ERK-mediated negative feedback loops on Ras and ErbB as representatives of a short- and long-feedback loop (Fig. 1a) in a nested feedback structure. We have previously demonstrated that individual feedback loops within coupled structures like this possess distinct dynamics-modulating function^74^, thus supporting the explicit modeling of two ERK-mediated loops.

#### FOXO3-mediated feedback loop

Both AKT and SGK1 phosphorylates FOXO3 at residues T32, S253 and S315, which promotes FOXO3 binding to 14-3-3 proteins and decreases its interaction with transcription coactivators CBP/p300^75,76^; note that SGK1 has a marked preference for Ser-315, whereas AKT favors Ser-253^76^. FOXO3 binds to S1/S4 on the *ESR1* promoter and induces ERα expression^77^.

#### ER-mediated feedback loops

The estrogen receptor (ER) plays a key role in the progression of breast cancer, particularly the luminal subtypes that encompass the T47D cell line. Once bound by estrogens, ER dimerizes and translocates to the nucleus where it interacts with transcriptional proteins^78^. In the model, we assumed that ERα induces the expression of c-Myc^79^ and promote the expression of SGK3^80,81^. ERα signaling is also regulated by other network components, including FOXO3 and ERK. FOXO3 is known to bind to the ERα promoter and enhance ERα signaling^77^; and ERK1/2 phosphorylates ERα at a number of serine sites which increases its activity^82^. These interactions are captured in our model, as displayed in Fig. 1a.

#### NDRG1-mediated feedback loop

In the model, we assumed that ErbB is negatively regulated by NDRG1 since NDRG1 enhances the interaction of ErbB with the ubiquitin ligase NEDD4^83,84^. Furthermore, NDRG1 is phosphorylated by SGK1/3 at T346/T356/T366, priming it for further phosphorylation by GSK-3β at S342/S353/S462^85,86^. Phosphorylation by GSK-3β is a common priming event for ubiquitination by the E3 ligase SCF complex Fbw7^86^. Thus, Fbw7 targets NDRG1 for degradation by the 26S proteasome^86^.

#### Other crosstalk mechanisms

There are multiple reciprocal crosstalk mechanisms between the PI3K/AKT and Ras/ERK pathways. In the model, we assumed that Ras directly activate PI3Kα through interacting via an amino-terminal Ras-binding domain (RBD)^69^. In the opposite direction, AKT phosphorylates and inhibits Raf, which leads to inhibition of the Raf/MEK/ERK cascade^87,88^, providing another crosstalk point between the two pathways. In addition, ERK phosphorylates GAB1 at six serine/threonine residues (T312, S381, S454, T476, S581, S597)^89^, which inhibit GAB1/PI3K association and thus suppresses the activity of PI3K^90^. ERK can also phosphorylate GAB2 and negatively regulates p85 recruitment^91^. Thus, in the model we assumed that ERK inhibits GAB, providing another crosstalk point between the two pathways.

### Model implementation

The new PI3K-centered model was formulated using ordinary differential equations (ODEs) following the interaction map depicted in Fig. 1a. The rate equations and full set of ODEs are given in Supplementary Tables 1 and 2. The model was implemented and numerically solved using MATLAB in conjunction with the IQM Tools (https://iqmtools.intiquan.com/) and the SUNDIALS suite (SUite of Nonlinear and DIfferential/ALgebraic equation Solvers, https://computing.llnl.gov/projects/sundials).

This combination of tools provides a scalable and powerful approach to construct and simulate ODE models of medium to large size, such as our model. Specifically: (1) an ODE function file containing definition of all model reactions, reaction rates and rate equations is created in IQM Tools based on the IQM syntax; (2) the ODE function file is transformed into C source code by IQM; (3) MATLAB then compiles and links the C code and SUNDIALS libraries into a binary MEX file; (4) and finally the MEX file is used for numerical solving by SUNDIALS’s ODE solver package CVODE. The main reason we employed SUNDIALS is because CVODE is significantly faster (~10 times) than conventional MATLAB solvers such as ODE15s in solving stiff and non-stiff ODE systems. Note that the speed may depend on a complexity and stiffness of the ODE systems. Essentially, the IQM toolbox provides an efficient MATLAB interface to SUNDIALS’s CVODE package.

### Model calibration and generation of “parental PI3K model”

Kinetic parameters in dynamic models are not determined experimentally and require calibration, or fitting, through experimental data to estimate unknown parameters. The potential discrepancy between simulated model output and experimental data can be minimized to find the best parameter values^92,93^. Model calibration is a critical step that provides a specific biological context to an otherwise ‘generic’ model. In this study, parameter estimation was performed by identifying the parameter set ***p*** in order to minimize the following ‘objective function’ that quantifies the mismatch between experimental measurements and corresponding simulated outputs^94^:

$$J({\boldsymbol{p}})=\mathop{\sum }\limits_{j=1}^{M}{\mathop{\sum }\limits_{i=1}^{N}\left(\frac{{y}_{j,i}^{D}-{y}_{j}({t}_{i},{\boldsymbol{p}})}{{\sigma }_{j,i}}\right)}^{2}$$

where *M* is the number of the experimental datasets used for fitting; *N* is the number of time points within each dataset; *y*_*j*_ (*t*_*i*_*,****p***) represents the numerically solved value of the model state variable *y*_*j*_ evaluated at time *t*_*i*_ and parameter set ***p***; while *y*^*D*^_*j,i*_ is the mean value of the corresponding data point at *t*_*i*_ with the associated variance of measured data *σ*_*j,i*_. Note that when averaged or single valued data are used, *σ*_*j,i*_ is set to be 1^95^.

#### Model calibration for T47D parental cells

We first generated a ‘parental model’ by performing model calibration using datasets obtained exclusively from the parental T47D breast cancer cell line. These include time-course and dose-response data of phosphorylated AKT and ERK, and expression of Cyclin D1 and p21 in response to stimulation by IGF-1, insulin and HRG, which were quantified and presented in Fig. 1b. ERK1/2 and AKT phosphorylation data by IGF-1 were downloaded from HMS LINCS Center (https://www.cancerbrowser.org/). ERK1/2 and AKT phosphorylation data and total level of cyclin D1, Myc and p21 by HRG were sourced from Neve et al., T47D cells^15^. Dose-response data of pAKT and pERK levels upon HRG stimulation were obtained from Neve et al., T47D cells^15^. Dose-response of pAKT, pERK and pS6 levels in T47D cells upon insulin stimulation were obtained from in-house experiments.

#### Genetic algorithm specification

We employed a genetic algorithm (GA) to optimize the objective function *J* due to its ability to avoid being trapped in local minima, use probabilistic selection rules, and based on our experience, work well for ODE models with a large number of parameters^96–98^. This was implemented using the Global Optimization Toolbox and the function *ga* in MATLAB. Selection rules select the individual solutions with the best fitness values (i.e., elite solutions) from the current population. The elite count was set to 5% of the population size. Crossover rules combine two parents to generate offspring for the next generation. The crossover faction was set at 0.8. Mutation rules apply random changes to individual parents to generate the population of the next generation. For the mutation rule, we generated a random number from a Gaussian distribution with mean 0 and standard deviation *σ*_*k*_, which was applied to the individuals of the current generation. The standard deviation function (*σ*_*k*_) is given by the recursive formula as follows:

$${\sigma }_{k}={\sigma }_{k-1}\left(1-\frac{k}{G}\right)$$

where *k* is the *k*th generation, *G* is the number of generations, and *σ*_0_ = 1.

#### Calibration implementation

Due to the size of our model, model calibration was carried out on a multi-processor virtual server consisting of 32 Intel Xeon 2.10 GHz processors running in parallel. To derive at the best fitted parameter set, we performed repeated GA runs with population size of 2000 and the generation number set to 100. During these runs, we also changed the mutation rate, crossover rate and even the population size in order to escape from being trapped in local minima^99^. After multiple repetitions of the GA process where the best fitted set obtained from a previous repeat was used as the starting point of the next repeat, we arrived at a best fitted set as the objective function was not further reduced, and the fitted parameter values no longer change.

#### Ensemble simulation to mitigate issues with model unidentifiability

Dynamic models in systems biology, particularly those of large size, face challenges of poor identifiability. This is primarily due to a lack of informative experimental data, and the existence of ‘local minima’ in the objective function landscape^100^. Thus, generally there is a trade-off between model identifiability and level of biological details. In this regard, our PI3K model suffers from unidentifiability because of its detailed scope and interactions, which were a deliberate design decision as we aimed to capture mechanistic feedbacks and crosstalk between multiple related pathways, and to facilitate a comprehensive prediction of drug response and drug combinations. To cope with this issue, we employed a two-pronged approach. First, we avoided the reliance (and possible biases) on a single best-fitted parameter set by repeating the GA-based procedure described above hundreds of time, each time with a different initial guess (generated from a log-uniform distribution in a range between −3 and 3), to obtain a final total of 77 independent best-fitted parameter sets that fitted the training data equally well (see Supplementary Fig. 1B and Supplementary Information). Critically, rather than using any single sets, we utilized all the obtained sets collectively for subsequent simulation and analysis, considering explicitly the mean and variance of simulated behaviors. This ‘ensemble’ strategy thus allows us to generate high-confidence predictions without strictly imposing identifiability on our model. Second, to gauge the calibrated model’s predictive power before making entirely new predictions, we further validated it with independent datasets that were not used during calibration (for example, see Supplementary Fig. 1A–D and relevant discussion in the main text). Together, our approach helps maximize the predictive capability of our parental model.

### Generation of “resistant PI3K model”

To generate a model describing the state of T47D cells resistant to BYL719 (called the resistant PI3K model) we comparatively profiled the basal (i.e., under standard growing condition) expression and/or phosphorylation levels of key network components between resistant and parental T47D cells. We next utilized this data to modify the basal signaling levels of the parental model and produced the resistant PI3K model. For example, our data showed that the expression levels of p21 and Cyclin D1 were about 7 and 3 folds higher in resistant cells than parental T47D cells, respectively; while the levels of AKT and ERK are relatively similar between the two cell types (Supplementary Fig 2C, D). Thus, in the resistant model, the initial concentrations of p21 and Cyclin D1 were adjusted accordingly to reflect the differential values in expression. This model customization strategy has been previously employed by us^95,101^ and others^102^, and proved to be a simple yet efficient strategy to specify models with the same network wiring to different biological contexts.

### Construction of the phenotypic model

Our data shows that BYL719 treatment causes DNA damage in T47D cells (Figs. 5 and 7d). Accumulation of DNA damage can prompt cells to initiate apoptosis and prevent proliferation of damaged cells. A crucial early step in this process involves the recruitment of the Mre11/Rad50/NBS1 (MRN) complex to double-stranded DNA break sites^103–105^. This action primes ATM activation, culminating in its auto-phosphorylation. Concurrently, ATR becomes active and similarly undergoes auto-phosphorylation. Once activated, both ATM and ATR phosphorylate a number of downstream targets, notably c-Abl (a non-receptor tyrosine kinase that phosphorylates the BH3-only protein Bim, countering the anti-apoptotic process) and specific caspases (namely caspase-8, -9, and -3)^106–108^.

For model simplicity, we assumed that DNA damage directly promoted apoptosis. The model also incorporates DNA damage-driven CHK1 activation, given that both ATM and ATR phosphorylate CHK1^109^. Once activated, CHK1 orchestrates the phosphorylation of diverse targets, including CDC25A, B and C, as well as p53^110^. This phosphorylation cascade inhibits CDC25A, B and C, blocking the transition to the G2/Mitosis phases. Concurrently, CHK1 also promotes DNA repair (R) by regulating the expression of DNA repair genes through activation of the transcription factor GADD45^111,112^. Importantly, Cyclin D1 and cyclin E, outputs from the mechanistic PI3K network model, drive the transition from G1 to S phase and act as inputs into the phenomenological model, thereby linking these two models to make up the new phonotypic models.

As with the original models, the phenotypic model was formulated using ordinary differential equations (ODEs) based on established kinetic laws^95,101^ (Supplementary Table 4). The model was then used to assess the impact of co-inhibiting PI3K with each of the 25 model nodes, including CHK1 as co-target, on cell viability. Cell viability was defined as the number of cells in DNA repair phase (R) or in G2/M phase (M), the latter emerging from successful DNA repair (Fig. 7d).

### Establishment of cell lines resistant to targeted therapies

The T47D human breast cancer cell line was purchased from the ATCC and cultured in RPMI1640 media supplemented with 10% fetal calf serum, 0.2 Units/ml of insulin, and 1% penicillin/streptomycin, as recommended. T47D cells were split in 1:3 to 1:4 twice a week. Cells with passage number lower than 20 were used in all experiments. To establish BYL719-resistant cell pools, T47D cells (200,000 per well in a 6-well plate) were exposed to increasing concentrations of BYL719 (Selleckchem, #S2814), starting with 1 μM and up to 30 μM, over 2 months. T47D resistant pool 1, 2 and 3 (herein RP1, RP2 and RP3) were generated and maintained in 1 μM BYL719, pulsed every 2 days. Similar procedures were used for the generation of T47D cells resistant to BKM-120 (Supplementary Fig. 5G).

For Saos2 cells, three independent plates of cells were chronically exposed to increasing concentrations of BYL719 (starting at 2 μM), or DMSO (similar v/v) for comparison. DMSO-treated cells reached confluency every 4–5 days, BYL719-treated cells remained quiescent for several weeks. However, after 6 weeks, BYL719-treated cells started to proliferate and to grow under selection.

### *CDKN1A* and Tp53 knock-down via CRISPR/Cas9

The Alt-R CRISPR-Cas9 System kit (Integrated DNA Technologies) was used to knock-down *CDKN1A* (p21) and TP53 in T47D cells according to the “Cationic lipid delivery of CRISPR ribonucleoprotein complex into mammalian cells” user guide Version 3. Briefly, a Cas9:crRNA:tracrRNA ribonucleoprotein (RNP) complex was generated by mixing equimolar concentrations of crRNA and tracrRNA stocks in nuclease-free duplex buffer, followed by the addition of the Cas9 enzyme and Cas9 PLUS reagent, mixed in Opti-MEM medium. Two independent crRNAs targeting *CDKN1A* (i.e., AB and AD) and one targeting Tp53 (i.e., AA) were used in this study. Transfection was performed by mixing 400,000 T47D RP cells with the RNP complex and lipofectamine 3000 in a 12-well plate. Cells were left undisturbed in a tissue culture incubator (37 °C, 5% CO_2_) for 48 h, after which complete RMPI medium with 10% FBS was used. *CDKN1A* knock-down was assessed by Western blotting using the indicated p21 and p53 antibody.

### Cell growth assays

T47D cells were plated in 24-well plates (25,000 cells/well) in full growth medium and left to attach overnight. The day after, one of the plates was washed in 1X PBS and fixed in 10% formalin at room temperature (RT) for 15 min, then washed with 1X PBS twice and stored at 4 °C until the end of the experiment (Day 0 plate). Remaining plates were treated as indicated in figures, and fixed on day 2, 4 and 6. At the end of the assay, cells were stained with crystal violet solution (0.1% (w/v) crystal violet in 20% methanol/dH_2_O at RT for 30 min. Each well was washed in 1X PBS three times and air dried overnight. Cell-bounded crystal violet was extracted in 10% (v/v) acetic acid solution for 30 min. Solutions were transferred to a 96-well plate and their absorbance measured at 590 nm using a PHERAstar FSX plate reader.

### Protein lysates and immunoblotting

T47D cells (150,000 cells/well) were plated in 6-well plates and treated as indicated. Standard protocols were used for western blotting analyses. At the end of each experiment, cells were washed in ice-cold PBS, scraped in RIPA lysis buffer (20 mM Tris-HCl pH7.8, 150 mM NaCl, 1% (v/v) NP-40, 0.05% (w/v) Sodium Deoxycholate, 0.4% (w/v) SDS, supplemented with 1X complete protease inhibitor (Roche) and 1X PhosSTOP (Roche)). Lysates were sonicated and cleared by centrifugation. Standard Laemmli-Buffer with 10% final concentration of β-mercaptoethanol was added and samples boiled for 5 min, resolved in NuPAGE Bis-Tris 4–12% protein gels (Life Technologies), and transferred onto nitrocellulose membranes. Membranes were blotted for 1 h in 5% (w/v) skimmed milk/ TBS-T (0.1% Tween 20 in Tris-buffered saline) and probed with the indicated primary antibodies in 5% (w/v) bovine serum albumin (BSA)/TBS-T overnight at 4 °C. After 3 washes in TBS-T buffer, membranes were probed with Amersham ECL horseradish peroxidase (HRP)-secondary antibodies (GE Healthcare) for 1 h at RT, followed by 3 washes in TBS-T buffer. Signal detection was performed by incubating membranes with Pierce ECL Western Blotting substrate followed by x-ray film development. All blots were derived from the same experiments and were processed in parallel. Uncropped scans of the most important blots are provided as Supplementary Figs. 7 and 8 in the Supplementary Information.

### 3D cell growth assay

Poly-2-hydroxethyl methacrylate (HEMA) solution (3% w/v) was prepared by dissolving poly-HEMA (Sigma-Aldrich, P3932) in 95% ethanol at 37 °C overnight. Wells of 24-well plates were coated with 400 μl poly-HEMA solution overnight. T47D cells (30,000/well) were plated and treated as indicated for 4 days. At the end of the treatment, cell aggregates were collected by centrifugation and dissociated with trypsin for cell counting.

### Proteomics

#### Culture of T47D cells and cell lysis

T47D cells, parental and resistant pools, were plated in 15 cm dishes, let to seed for 48 h and then treated with 1 μM BYL719 for 24 h before harvest. Cells were scraped off in growth medium and spun at 250 × *g* for 10 min at RT. Cells were washed with 1X PBS at 320 × *g* for 7 min, cell pellets were lysed in RIPA buffer containing protease inhibitor (Roche) and 1X PhosSTOP (Roche, in HPLC-grade water) on ice for 5 min, and then sonicated and cleared at 4 °C by centrifugation.

#### Protein precipitation and digestion

Cell lysates were precipitated in 5X volume of ice-cold acetone at −20 °C overnight. Protein precipitates were spun at 17,000 × *g* for 10 min at 4 °C and washed once in ice-cold acetone. Proteins were re-solubilised in 8 M urea buffer containing 10 mM tris(2-carboxyethyl) phosphine (TCEP) assisted by sonication. Protein concentration was quantified and samples normalized to the same volume. Samples were then alkylated in 55 mM iodoacetamide for 45 min in the dark. Urea in samples was diluted to 1 M with 25 mM triethylammonium bicarbonate (TEAB), pH 8.0 before digestion with Pierce™ Trypsin Protease, MS Grade (Thermo Fisher Scientific, #90059) at 37 °C overnight. Samples were then acidified with formic acid to a final concentration of 1% (v/v).

#### Samples’ clean-up and phosphopeptide enrichment

Solid-phase extraction (Oasis HLB, Waters, #WAT094226) was used for peptides cleaning. Briefly, cartridges were conditioned with 80% (v/v) acetonitrile/0.1% (v/v) trifluoroacetic acid (TFA) followed by equilibration with 0.1% TFA. Samples were then loaded and allowed to bind to the sorbent in the columns. Columns were then washed twice with 0.1% TFA and peptides eluted in 80% (v/v) acetonitrile/0.1% (v/v) TFA. Samples were freeze-dried overnight. Samples were then resuspended in loading buffer (2 M lactic acid in 50% acetonitrile and 5% TFA) through vortexing and sonication. Titansphere, spherical TiO_2_ beads (GLSciences, 5020-75000) at a ratio of 6 mg/mg proteins were conditioned by washing in Washing buffer (50% acetonitrile and 5% TFA) followed by Loading buffer. Samples were incubated with 80% of the conditioned TiO_2_ beads for 60 min with 450 × *g* shaking and spun down. Supernatants were incubated with remaining TiO_2_ beads for an extra 30 min. After TiO_2_ enrichment, beads were packed into a home-made stage tip (a C8 plug in a 200 μl tip). Peptide samples were loaded into the tips and spun through the tips at 1250 × *g* for 10 min. Tips were then washed with Loading buffer and Washing buffer until all samples were loaded. Phosphopeptides were eluted in 1% (v/v) ammonium hydroxide followed by 30% (v/v) acetonitrile. Samples were freeze-dried overnight.

#### Data-dependent acquisition (DDA) LC-MS/MS

Samples were analyzed by LC-MS/MS using Orbitrap Lumos mass spectrometer (Thermo Scientific) fitted with nanoflow reversed-phase-HPLC (Ultimate 3000 RSLC, Dionex). The nano-LC system was equipped with an Acclaim Pepmap nano-trap column (Dionex—C18, 100 Å, 75 μm × 2 cm) and an Acclaim Pepmap RSLC analytical column (Dionex—C18, 100 Å, 75 μm × 50 cm). Typically for each LC-MS/MS experiment, 10 μl of the peptide mix was loaded onto the enrichment (trap) column at an isocratic flow of 5 μl/min of 3% CH3CN containing 0.1% formic acid for 8 min before the enrichment column is switched in-line with the analytical column. The eluents used for the LC were 5% DMSO/0.1% v/v formic acid (solvent A) and 100% CH3CN/5% DMSO/0.1% formic acid v/v (solvent B). The gradient used was 3% B to 25% B for 177 min, 20% B to 40% B in 5 min, 40% B to 80% B in 5 min and maintained at 80% B for the final 5 min before equilibration for 10 min at 3% B prior to the next analysis.

The mass spectrometer was operated in positive-ionization mode with spray voltage set at 1.9 kV and source temperature at 275 °C. Lockmass of 401.92272 from DMSO was used. The mass spectrometer was operated in the data-dependent acquisition mode MS spectra scanning from *m*/*z* 350–1550 at 120,000 resolution with AGC target of 5e5. The “top speed” acquisition method mode (3 s cycle time) on the most intense precursor was used whereby peptide ions with charge states ≥2–5 were isolated with isolation window of 1.6 *m*/*z* and fragmented with high energy collision (HCD) mode with stepped collision energy of 30 ± 5%. Fragment ion spectra were acquired in Orbitrap at 15,000 resolution. Dynamic exclusion was activated for 30 s.

#### Protein identification and quantification

MS data from DDA profiling were processed using Maxquant v1.5.5.1. MS/MS spectra were searched against the Uniprot human reference proteome FASTA file (downloaded on 21 February 2017). Digestion mode was set as trypsin. Label-free quantification (LFQ) was applied. Carbamidomethyl cysteine was set as a fixed modification; oxidation of methionine, phosphorylation of serine, threonine and tyrosine were considered variable modifications. False discovery rate (FDR) was set to 0.01.

#### Data analysis

Statistical analysis was performed using the Perseus package (Max Planck Institute of Biochemistry)^113^. A localization probability of ≥0.75 to a single amino acid residue for phosphopeptide was applied and parameters were set as default. Differential expression between groups was analyzed by two-sample *t*-test with the significance cut-off being *p* < 0.05. Pathway enrichment analysis was performed using the Ingenuity Pathway Analysis (IPA) package (QIAGEN Inc., https://digitalinsights.qiagen.com/IPA) using default settings.

### Cell cycle analysis

T47D cells (150,000 cells/well) were plated in 6-well plates and treated as indicated. One hour before the end of treatment, cells were pulsed with 1:100 BrdU (Invitrogen #00-0103). For cell cycle analysis, cells were trypsinised, pelleted by centrifugation and ice-cold 70% ethanol was added dropwise into each sample while on gentle vortex. After fixing on ice for 1 h, cells were washed twice in PBS at 500 × *g* for 10 min. 2 N HCl/0.5% Triton X-100 was added dropwise to permeabilise and denature the DNA at RT for 30 min. The pH was neutralized with 0.1 M sodium tetraborate decahydrate NaB_4_O_7_ solution (pH8.5) and samples washed in 1%BSA/PBS. Cells were labeled with anti-BrdU primary antibodies (abcam #ab6326) and secondary antibodies in 1% BSA/0.2% Tween-20 in PBS. Samples were resuspended in propidium iodide/RNase solution. Cell cycle profiles were analyzed using a BD LSRFortessa^TM^ X-20 flow cytometer. Raw data were analyzed using FlowJo 10.3.0 software package.

### Quantitative reverse-transcription PCR

Cells were lysed in TRI reagent (Thermo Fisher Scientific, #AM9738) and total RNA extracted using Direct-zol RNA Miniprep Kits (Zymo Research, #R2050). RNA was reverse-transcribed into cDNA with QuantiTect Reverse Transcription Kit (QIAGEN). qPCR was performed in 2X QuantiNova STBR Green PCR Master Mix in CFX384 Touch Real-Time PCR Detection System (Bio-Rad). Ct values were determined in Bio-Rad CFX Manager v3.1 software package and the relative quantification was derived using the ΔΔCt method.

### β-galactosidase cell senescence assay

T47D cells (25,000 cells/well) were plated on collagen-coated glass coverslips (10 μg/cm^2^) in 24-well plates overnight and treated as indicated. Drugs were pulsed every 2 days and at the end of treatments, cells were washed with 1X PBS and processed according to the senescence β-galactosidase (β-gal) staining kit (Cell Signaling Technologies, #9860). Cells were then counter-stained with DAPI (1 μg/ml) and mounted in 70% glycerol on glass slides. Olympus dotSlide digital virtual microscope was used to acquire DAPI and bright-field images. Total cell number (DAPI) and β-gal positive cells were quantified using Fiji software package.

### γH2AX and 53BP1 staining

T47D cells (200,000 cells/well) were plated in 6-well plates and treated as indicated. At the end of each treatment, cells were treated with KaryoMAX Colcemid at 10 mg/ml for 1 h prior to harvest. Cells were subjected to hypotonic treatment in 0.075 M KCl (room temperature for 5 min), cyto-spun onto slides, and incubated in ice-cold KCM buffer (120 mM KCl, 10 mM Tris-HCl pH 7.5, 20 mM NaCl, 0.5 mM EDTA, 0.1% (v/v) Triton X-100 and protease inhibitor) for 5 min. Slides were incubated in ice-cold KCM extraction buffer (KCM and 0.4% Triton X-100) for 5 min, followed by an incubation in ice-cold KCM blocking buffer (KCM, 2% BSA, protease inhibitor and AEBSF) for another 5 min. Slides were incubated in anti-centromere human CREST (calcinosis, Raynaud phenomenon, esophageal dysmotility, sclerodactyly, and telangiectasia) and anti-phospho-histone H2A.X (Ser139) (Merck Millipore JBW301), or anti-53BP1 (ab21083) and secondary antibodies for 1 h at 37 °C in KCM block buffer. After antibody incubation, slides were washed three times with ice-cold KCM, and then fixed in 4% (v/v) formaldehyde (in KCM) and mounted with DAPI in Vectashield media. Images were collected using a Zeiss imager M2 fluorescence microscope linked to an AxioCam MRm CCD camera system. Image analysis was performed in Fiji software package. DAPI stain was used to create a mask for nuclei. Integrated fluorescence intensity of γH2AX per nucleus and 53BP1 per nucleus was quantified.

### Cell death assay

T47D cells (25,000 cells/well in 24-well plate) were incubated with propidium iodide (PI) (1 mg/ml) (Thermo Fisher Scientific, P3566) and Hoechst 33342 solution (0.5 mg/ml) (Thermo Fisher Scientific, #62249) in a cell culture incubator for 30 min. Multiple fields per wells were imaged using a Leica DMi8 inverted microscope at ×10 magnification. Fiji software package was used to quantify the number of PI^+^ cells and the total cell number per field.

### p21 cellular localization

T47D cells were grown on collagen-coated cover slips (25,000 cells/well in a 24-well plate) and treated as indicated. Cells were then fixed in 4% formaldehyde for 15 min and washed with 1X PBS three times. Permeabilisation and blocking step was done by incubating cells in 5% normal goat serum with 0.3% Triton-X 100 for 1 hr at RT. Standard IF protocol was used to probe for p21 using the rabbit mAb CST#2947. Cells were stained with DAPI and Wheat Germ Agglutinin-Alexa Fluor 555 for nuclear and whole cell segmentation, respectively. Multiple fields per well were imaged using a Leica DMi8 microscope at 20X magnification. Integrated intensity of p21 localization in nuclei and whole cells was quantified using the CellProfiler software package.

### CellTiter-Glo assay

The dose-response of T47D cell lines to small molecule inhibitors was assayed using the CellTiter-Glo® 3D-Cell Viability assay (Promega, #G9681) according to the manufacturer’s instructions. Briefly, 5000 cells/well were plated in a 96-well plate and treated with increasing concentrations of inhibitors. At the end of each treatment, culture media was removed and plain RPMI media was added to each well. An equal volume of CellTiter reagent was added to the wells and plate/s shaken in a PHERAstar FSX for 5 min. Plates were then incubated in the dark for 30 min on a platform rocker, at the end of which contents were transferred into white OptiPlates and the integrated luminescence signal detected using a PHERAstar FSX.

### Simulation of drugs combinations and drugs synergy

Drug synergy was computed based on coefficients of drug interaction (CDI) metric^17,114^: CDI = *E*_12_/(*E*_1_ × *E*_2_), where *E*_12_ is the normalized effect induced by drug 1 combined with drug 2, on a specific biological readout; *E*_1_ and *E*_2_ represent the effect of a single drug. CDI values lower than 1 indicate synergistic effects, CDI values equal or higher than 1 indicate additive or antagonistic effects, respectively. The degree of synergism versus antagonism is indicated by how small or large CDI values are compared to 1.

### Patient survival analysis

mRNA expression, mutation profile and associated overall survival (OS) data from 2509 breast cancer patients (METABRIC)^115^ were downloaded from cBioPortal (https://www.cbioportal.org/). Breast cancer patients were classified based on *CDKN1A* and *CCND1* expressions into three groups having either low, normal, or high expression levels. These were associated with the *PIK3CA* mutational status defined as either mutated or wildtype. Combination sub-groups were derived as such: (i) PIK3CA mutation and *CDKN1A* (or *CCND1*) high; (ii) *PIK3CA* mutation and *CDKN1A* (or *CCND1*) low; (iii) *PIK3CA* wildtype and *CDKN1A* (or *CCND1*) high; (iv) *PIK3CA* wildtype and *CDKN1A* (or *CCND1*) low. OS between sub-groups were subsequently performed using R package ‘survival’, with *p* < 0.05 considered significant.

**List of antibodies with catalog numbers and dilutions:**

**Table:** 

Rabbit monoclonal anti-Phospho-Akt (Ser473) (clone D9E) 1:1000 dilution	Cell Signaling Technology	Cat# 4060; RRID:AB_2315049
Rabbit monoclonal anti-Phospho-Akt (Thr308) (clone D25E6) 1:1000 dilution	Cell Signaling Technology	Cat# 13038; RRID:AB_2629447
Rabbit monoclonal anti- Phospho-S6 Ribosomal Protein (Ser240/244) (clone D68F8) 1:2000 dilution	Cell Signaling Technology	Cat# 5364, RRID:AB_10694233
Rabbit monoclonal anti-S6 Ribosomal Protein (clone 5G10) 1:2000 dilution	Cell Signaling Technology	Cat# 2217; RRID:AB_331355
Mouse monoclonal anti-Actin (clone AC-40) 1:5000 dilution	Sigma-Aldrich	Cat# A3853; RRID:AB_262137
Rabbit monoclonal anti-Phospho4E-BP1 (Thr37/46) (236B4) 1:1000 dilution	Cell Signaling Technology	Cat# 2855; RRID:AB_560835
Rabbit monoclonal anti-Akt (pan) (clone 11E7) 1:1000 dilution	Cell Signaling Technology	Cat# 4685; RRID:AB_2225340
Rabbit monoclonal anti-Phospho Rb (Ser807/811) 1:2000 dilution	Cell Signaling Technology	Cat# 8516; RRID:AB_11178658
Rabbit monoclonal anti-Phosphop44/42 MAPK (Erk1/2) (Thr202/Tyr204) 1:1000 dilution	Cell Signaling Technology	Cat# 4370; RRID:AB_2315112
p44/42 MAPK (Erk1/2) 1:1000 dilution	Cell Signaling Technology	Cat# 4696; RRID: AB_390780
Rabbit polyclonal anti-Cyclin D1 1:1000 dilution	Cell Signaling Technology	Cat# 2922; RRID:AB_2228523
Mouse monoclonal anti-Cyclin A (clone AT10.2) 1:1000 dilution	Santa Cruz Biotechnology	Cat# sc-53227; RRID:AB_782329
Rabbit monoclonal anti-CDK4 (clone D9G3E) 1:1000 dilution	Cell Signaling Technology	Cat# 12790; RRID:AB_2631166
Mouse monoclonal anti-CDK6 (clone DCS22) 1:1000 dilution	Cell Signaling Technology	Cat# 3136; RRID:AB_2229289
Rabbit monoclonal anti-CDK2 (clone 78B2) 1:1000 dilution	Cell Signaling Technology	Cat# 2546; RRID:AB_2276129
Rabbit monoclonal anti-p21 Waf1/Cip1 (clone 12D1) 1:1000 dilution	Cell Signaling Technology	Cat# 2947 RRID:AB_823586
Mouse monoclonal anti-p18 INK4C (clone DCS118) 1:1000 dilution	Cell Signaling Technology	Cat# 2896; RRID:AB_331203
Rabbit monoclonal anti-p27 Kip1 (clone D69C12) 1:1000 dilution	Cell Signaling Technology	Cat# 3686; RRID:AB_2077850
Rabbit monoclonal anti-Phospho-Chk1 (Ser345) (clone 133D3) 1:1000 dilution	Cell Signaling Technology	Cat# 2348; RRID:AB_331212
Mouse monoclonal anti-PCNA (clone PC10) 1:1000 dilution	Cell Signaling Technology	Cat#2586; RRID:AB_2160343
Rabbit monoclonal anti-CDKN2A/p16INK4a (clone EPR1473) 1:1000 dilution	Abcam	Cat# ab108349; RRID:AB_10858268
Mouse monoclonal anti-p53 (clone 1C12) 1:1000 dilution	Cell Signaling Technology	Cat# 2524; RRID:AB_331743
Rabbit polyclonal anti-53BP1 1:500 dilution	Abcam	Cat# ab21083 RRID:AB_722496
Mouse monoclonal anti-phospho-Histone H2A.X (Ser139) (clone JBW301) 1:1000 dilution	Millipore	Cat# 05-636, RRID:AB_309864
Rabbit monoclonal anti-phospho-NDRG1 (Thr346) (D98G11) XP® 1:500 dilution	Cell Signaling Technology	Cat# 5482; RRID:AB_10693451
Rabbit monoclonal anti-hospho-SGK3 (Thr320) (D30E6) 1:500 dilution	Cell Signaling Technology	Cat# 5642; RRID: AB_10694357

Uncropped scans of the most important blots and gating strategy for Fig. 3d are provided as Supplementary Figs. 7–9 in the Supplementary Information File.

### Reporting summary

Further information on research design is available in the Nature Research Reporting Summary linked to this article.

### Supplementary information

Supplementary Information

Supplementary Dataset 1

Supplementary Dataset 2

Supplementary Dataset 3

REPORTING SUMMARY

## Data Availability

Proteomics data have been deposited to the ProteomeXchange Consortium via PRIDE^1^ partner repository with the dataset identifier PXD033956.

## Code availability

All the codes for modeling have been deposited in Github and can be accessed at: https://github.com/NguyenLabNetworkModeling/Integrated-PI3K-Signaling-Network-model.
