# Supplementary material for: Integrative modeling uncovers p21-driven drug resistance and prioritizes therapies for PIK3CA-mutant breast cancer
Source: NPJ Precis Oncol. 2024 Jan 26;8:20. doi: 10.1038/s41698-024-00496-y (PMC10810864; doi:10.1038/s41698-024-00496-y)
Supplement: Supplementary file 1 — Supplementary Information [file 41698_2024_496_MOESM1_ESM.pdf]

Supplementary Information for:

"Integrative modelling uncovers p21-driven drug resistance and prioritizes therapies for PIK3CA-mutant breast cancer"

Hon Yan Kelvin Yip[1,2], Sung-Young Shin[1,2], Annabel Chee[1,3], Ching-Seng Ang[4], Fernando J Rossello[5,6,7,8], Lee Hwa Wong[1], Lan K. Nguyen[1,*], and Antonella Papa[1,9,*]

[1]Cancer Program, Monash Biomedicine Discovery Institute and Department of Biochemistry and Molecular Biology, Monash University, Melbourne, Victoria 3800, Australia.

[4]Bio21 Mass Spectrometry and Proteomics Facility, The University of Melbourne, Parkville, VIC 3010, Australia.

[5] Murdoch Children's Research Institute, The Royal Children's Hospital, Melbourne, VIC 3052, Australia

[6]Novo Nordisk Foundation Center for Stem Cell Medicine, Murdoch Children's Research Institute, Melbourne, VIC 3052, Australia

[7]Department of Clinical Pathology, University of Melbourne, Melbourne, VIC, Australia

[8]Australian Regenerative Medicine Institute, Monash University, Victoria, Australia.

[2]These authors contributed equally.

[3]*Present address:* Centre for Muscle Research, Department of Anatomy and Physiology, The University of Melbourne, Melbourne, VIC 3010, Australia

[9]Lead contact

[*]Corresponding author: lan.k.nguyen@monash.edu (L.K.N), antonella.papa@monash.edu (A.P.)

This PDF file includes:

Supplementary Figures 1 to 9

Supplementary Tables 1 to 4

Other supplementary materials for this manuscript include the following:

Dataset 1. List of best-fitted parameter values used for ensemble model calibration.

Dataset 2. CDI scores and effect of single and combination drugs shown in Fig. 2A-F and Supplementary Fig. 3C, D.

Dataset 3. Parameters and constants for the phenotypic model.

Supplementary Figures

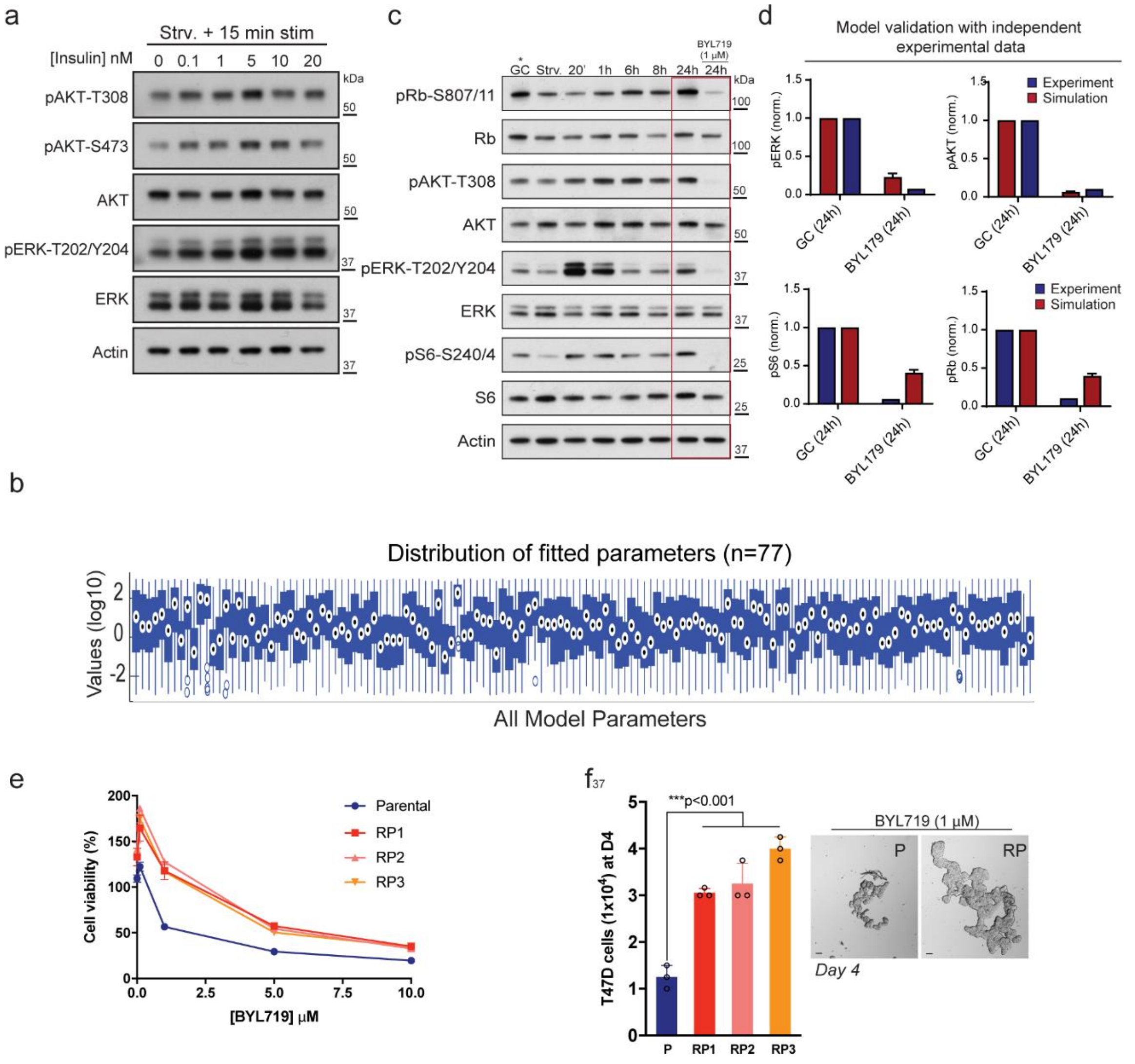

Supplementary Figure 1. (a) Parental T47D cells were starved for 24 hrs in DMEM, and stimulated for 15 min with insulin at the indicated concentrations. **(b)** Box plot of fitted parameters (n=77). **(c)** WB of parental T47D cells kept under growing conditions (GC) or starved for 24 hrs in DMEM and then stimulated with full medium for the indicated times. One well of T47D cells was stimulated with full medium plus 1µM BYL719 for 24h to test response to treatment. **(d)** Model validation using independent experimental data, quantified from WB images shown in (c). Red and blue bars indicate simulation and experimental data, respectively. Data represent mean values and error bars indicate standard errors (n=77). Effect of BYL719 on ERK, AKT, S6 and RB phosphorylation. **(e)** Cell viability assay of

parental and resistant T47D pools (RPs) grown under increasing concentration of BYL719, i.e. 0.1, 1, 5 and 10 μM for 4 days, and processed with the CellTiter-Glo® 3D-Cell Viability assay. Data are represented as mean ± standard deviation (SD) of triplicate wells (n=3). **(f)** 3D growth curve of parental T47D cells and BYL719-RPs plated on p(HEMA) coated dishes and kept in full medium plus 1 μM BYL719 for 4 days. Right, representative images of parental (P) and resistant pool 1 (RP) 4 days of treatment. Data are represented as mean ± SD, one-way ANOVA, (n=3 replicates of culture), ***p<0.001.

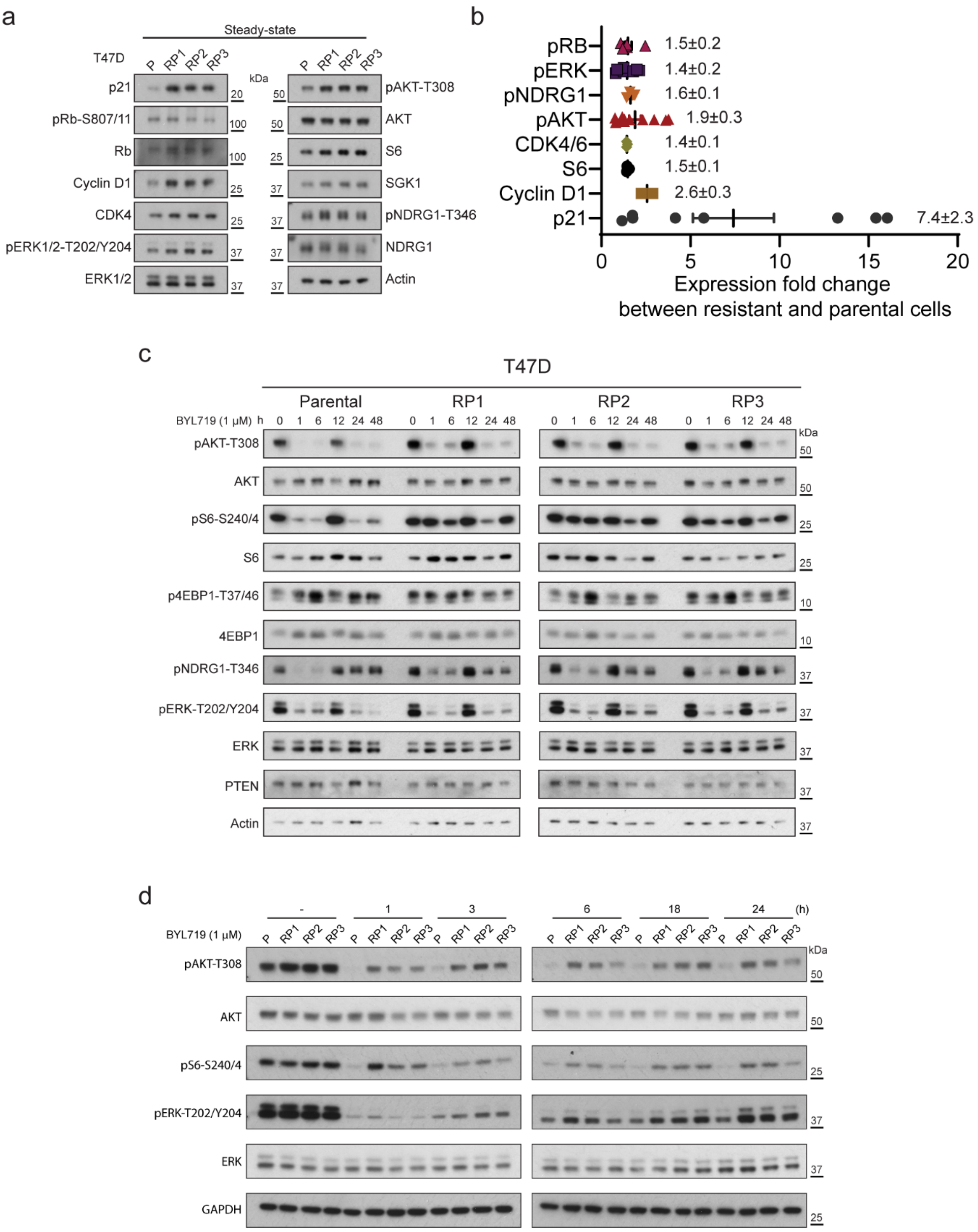

Supplementary Figure 2. (a) Representative cell lysates of parental and T47D RPs cultured in growing condition. Expression/phosphorylation of multiple network nodes was analysed by WB. **(b)** Relative fold change in expression and/or phosphorylation of signalling proteins in RPs compared to parental cells, quantified from WB data (as in panel a). Data was quantified from 3 independent experiments as mean ± standard error.

(c) Parental T47D cells and three BYL719- RPs were either left untreated or treated with BYL719 (1 μM) for 1, 6, 12, 24 and 48h or **(d)** 1, 3, 6, 18 and 24h. Cell lysates were analysed by WB **(**See also Figure 1G).

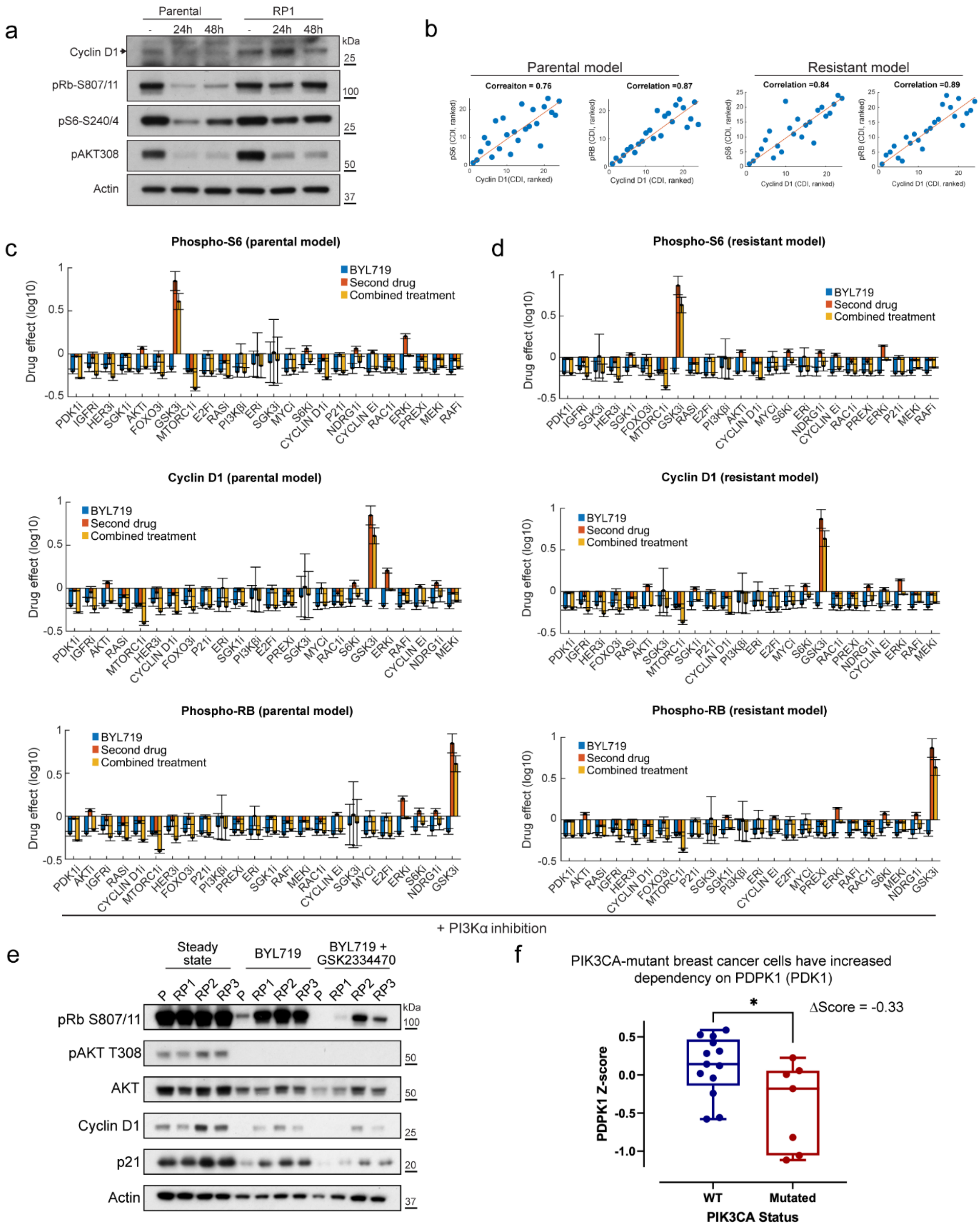

Supplementary Figure 3. (a) WB analysis of cyclin D1 and pRb in lysates of T47D parental and RP1 treated with BYL719 for the time indicated. **(b)** Correlation analysis of the synergy scores between readouts, i.e., pS6, Cyclin D1 and pRb. Effect of single and combination drugs treatments on pS6, cyclin D1 and pRb levels in parental **(c)** and resistant PI3K models **(d)**. Bars indicate the mean values and ± standard errors (see Dataset 2). **(e)** WB analysis of

p21 and cell cycle regulators in lysates of T47D cells. T47D parental cells and RP1 were treated with 1 μM BYL719 or 0.5 μM of the PDK1 inhibitor GSK2334470, either alone or in combination, for 24 and 48h. **(f)** Dependency scores of the *PDPK1* gene in *PIK3CA*-mutant (n=13) and -WT (n=7) breast cancer cells, DEPMAP; note that a lower negative score value indicates stronger dependency, $*p<0.05$.

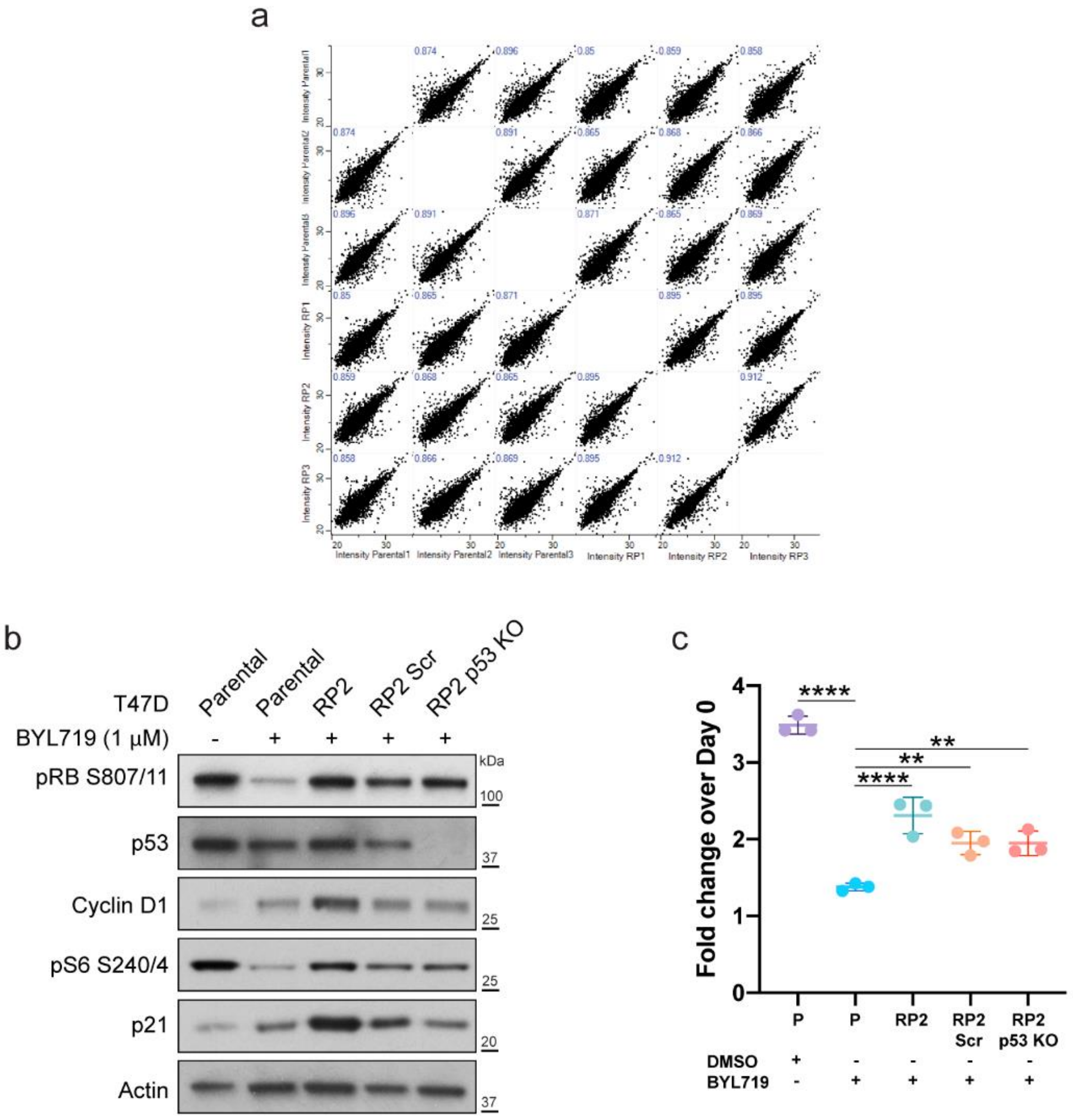

Supplementary Figure 4. (a) Pearson's correlation of phosphopeptides identified in triplicate samples of T47D parental cells and RP1, RP2 and RP3. **(b)** WB showing successful *Tp53 knock-out* (KO) in BYL719-RP2 cells using CRISPR. RP2 Scr samples are cells transduced with negative scramble (Scr) control. **(c)** Comparative growth curves at day 6 of the indicted cell lines upon 1μM BYL719 treatment. Data are presented as mean ± SD, one-way ANOVA, (n=3 replicates of culture) **p<0.01, ****p<0.0001.

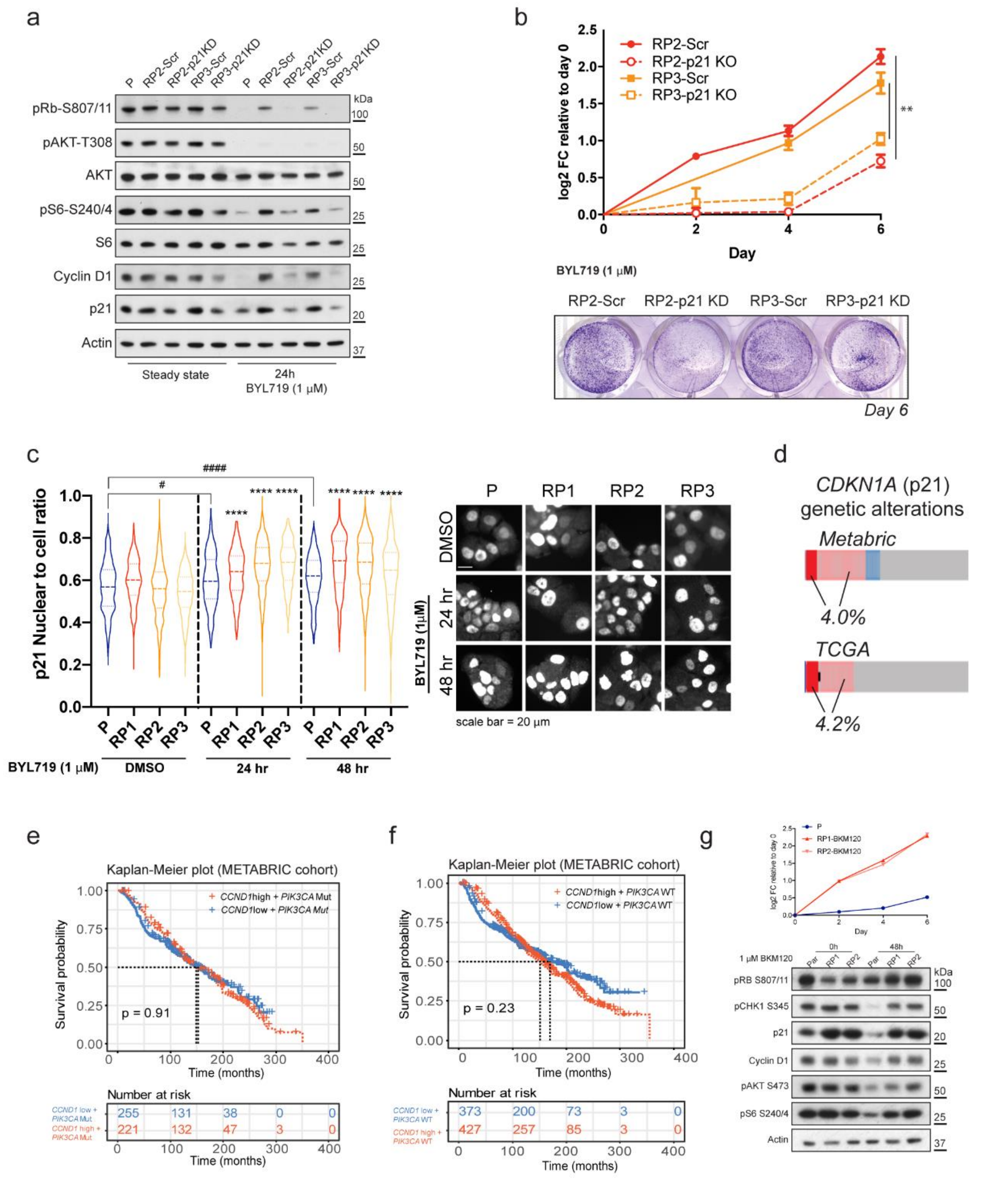

Supplementary Figure 5. (a) WB analysis of cell cycle regulators in T47D cells. CRISPR/Cas9 approach was used to transduce T47D RP1 cells with scramble (Scr) or 2 independent guide-RNAs directed at *CDKN1A* (p21KD-1, -2). Cells were either left untreated or treated with 1 μM BYL719 for 24h. **(b)** *CDKN1A* knock-down re-sensitises T47D RP1 cells to BYL719. T47D cells were treated with 1 μM BYL719 for 6 days and new drug added to the medium every 2 days. Representative images of cells stained with crystal violet on day

6 (bottom). Data are presented as mean ± SD, one-way ANOVA, (n=3 replicates of culture) **$p<0.01$. **(c)** Parental (P) and resistant T47D cells were treated with 1 μM BYL719 for 24 and 48 hrs, and fluorescence intensities quantified using CellProfiler. The fluorescence intensity of nuclear p21 was normalised over the intensity detected in the entire cell, and results are presented as cell ratio. Data represent median and the 1st and 3rd quartile. ####$p<0.0001$ indicate comparisons between the same cell type upon BYL719 treatments compared to DMSO; ****$p<0.0001$ are comparisons between RPs and parental in the same treatment condition. **(d)** Percentage of genetic alterations affecting *CDKN1A* in human breast cancer samples (Metabric and TCGA datasets). Alterations legends: deep deletion (dark blue), amplification (red), high mRNA levels (pink), low mRNA level (blue), black (truncating mutations) and grey (unaltered). **(e, f)** Probability of overall survival (OS) of breast cancer patients (METABRIC). **(g)** *Top*: growth curves of parental and BKM-120-resistant T47D cells over 6 days of treatments in 1 μM BKM-120. *Bottom*: WB showing markers of resistance in parental and BKM-120-resistant T47D cells.

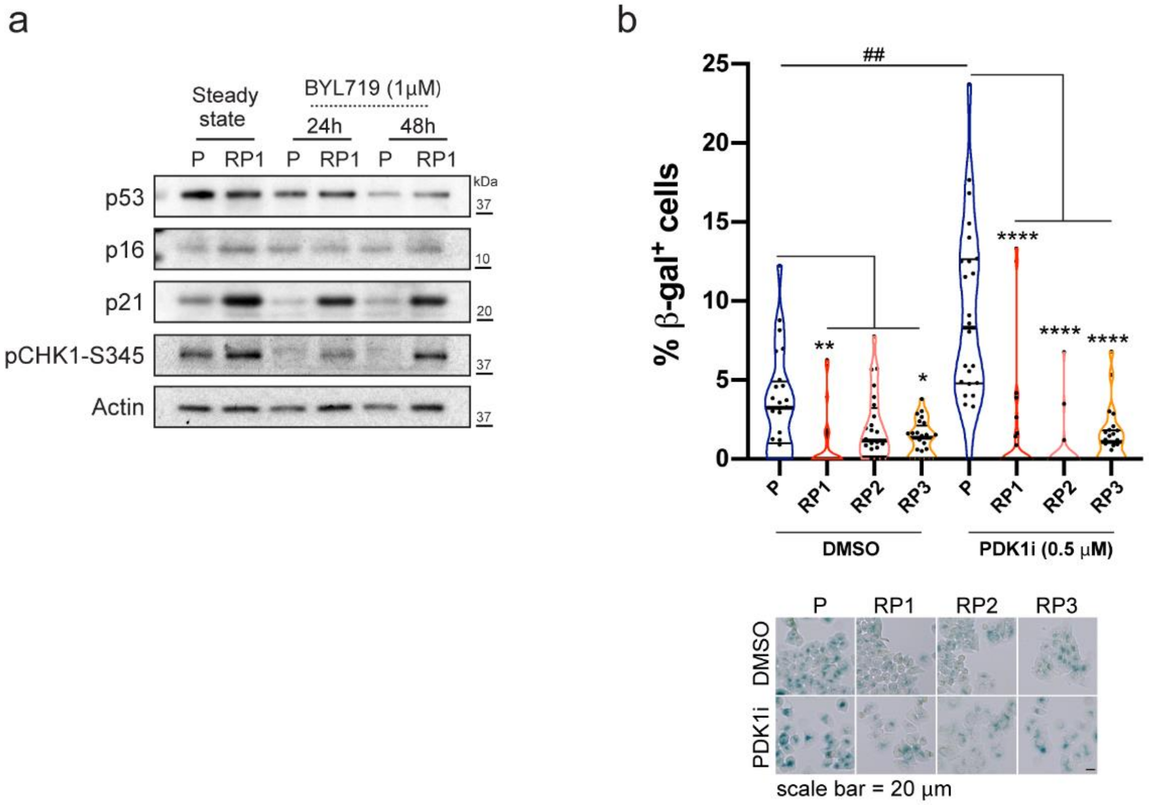

Supplementary Figure 6. (a) WB analysis of senescence regulators in T47D parental and RP1 cells treated with 1μM BYL719 for 24 and 48h. **(b)** T47D parental and RPs were treated with 0.5 μM GSK2334470 for 48hrs, then fixed and stained to detect β-gal positivity. ##p<0.01, *p<0.05, **p<0.01, ****p<0.0001.

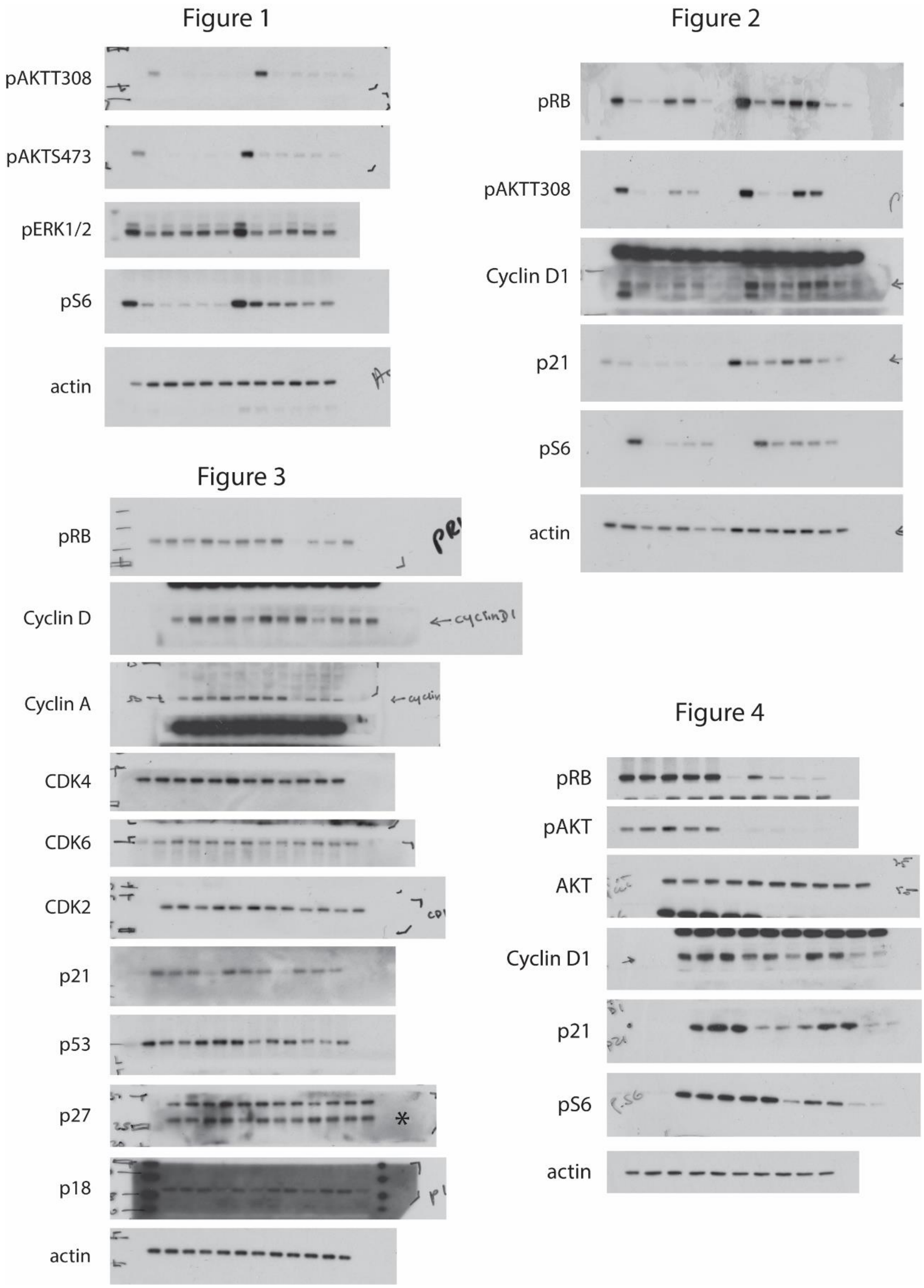

Supplementary Figure 7. Uncropped scans of blots included in main figures 1 to 4.

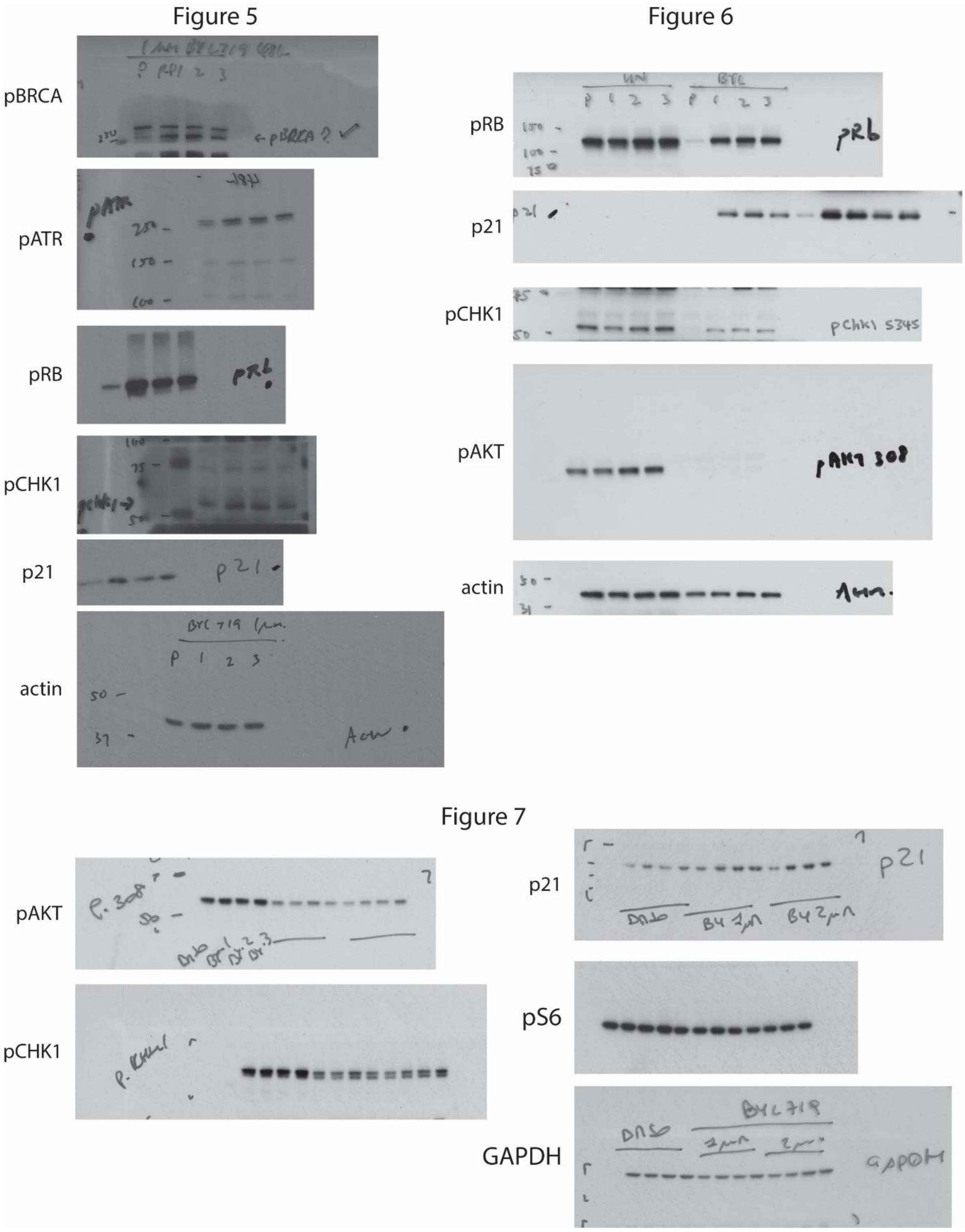

Supplementary Figure 8. Uncropped scans of blots included in main figures 5 to 7.

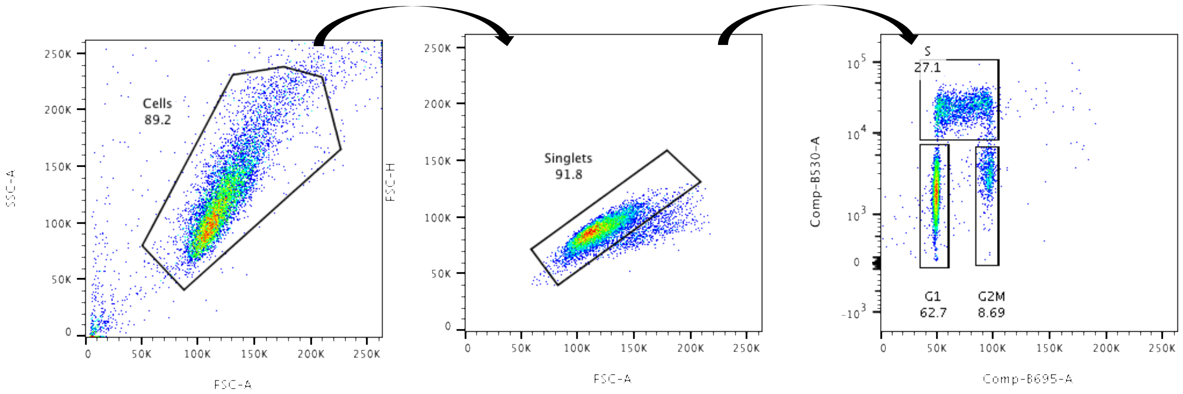

Supplementary Figure 9. Gating strategy used for cell cycle analysis in Figure 3d. Upon treatments, T47D cells were gated by the “Cells” gate in the SSC-A vs FSC-A plot. Single cells, excluding doublets, were then gated in the “Singlets” gate in the FSC-H vs FSC-A. The distribution of cell cycle phases was analysed on intact single cells by BrdU-FITC antibodies (Comp-B530-A) and propidium iodide (Comp-B695-A).

Supplementary Tables

Supplementary Table 1.

Reactions and reaction rates of the Integrated PI3K signaling network model.

	Reaction	Reaction rates
rt01	IGF + IR → IRL	ka0*Ins*IR - kd0*IRa
rt02	IRL → IRp	kc1_rt_002 * IRL - vm_rt_002 * IRp
rt03	IRp → IRi	kc1_rt_003 * IRp - vm_rt_003 * IRi
rt04	PI3Kα → IRp_PI3Kα	kc2_rt_004 * IRp * PI3Kα /(1 + Ki_rt_004*pS6K) - vm_rt_004 * IRp_PI3Kα
rt05	PI3Kβ → IRp_PI3Kβ	kc2_rt_005 * IRp * PI3Kβ /(1 + Ki_rt_004*pS6K) - vm_rt_005 * IRp_PI3Kβ
rt06	PI3Kα → pHER3L_PI3Kα	kc2_rt_006 * pHER3L * PI3Kα - vm_rt_006 * pHER3L_PI3Kα
rt07	PI3Kβ → pHER3L_PI3Kβ	kc2_rt_007 * pHER3L * PI3Kβ - vm_rt_007 * pHER3L_PI3Kβ
rt08	PIP2 → PIP3	PIP2*kc2_rt_008/(Km_rt_008 + PIP2)*((mtPIK3CA + (IRp_PI3Kα + pHER3L_PI3Kα)*(1+alpha_rt_008a*mGABp + alpha_rt_008b*tRas))*(1-w_drg*BYL719_on*BYL719/(Ki_drg + BYL719)) + (IRp_PI3Kβ + pHER3L_PI3Kβ)*(1+alpha_rt_008a*mGABp + alpha_rt_008c*tRac1))
rt09	PIP3 → PIP2	kc2_rt_009 * PIP3 * PTEN
rt10	GAB → mGABp	kc2_rt_010 * GAB * PIP3 / (1 + Ki_rt_010*pErk) - vm_rt_010 * mGABp
rt11	PDK1 → mPDK1	kc2_rt_011 * PDK1 * PIP3 - vm_rt_011 * mPDK1
rt12	PREX1 → mPREX1	kc2_rt_012 * PREX1 * PIP3 - vm_rt_012 * mPREX1
rt13	dRac1 → tRac1	kc2_rt_013 * mPREX1 * dRac1 - vm_rt_013 * tRac1
rt14	Akt → pAkt	kc2_rt_014 * mPDK1 * Akt /(1 + ki_rt_014*ppRB) - vm_rt_014 * pAkt
rt15	SGK1 → pSGK1	kc2_rt_015 * SGK1 * mPDK1 /(1 + ki_rt_014 * ppRB) - vm_rt_015 * pSGK1
rt16	SGK3 → pSGK3	kc2_rt_016 * mPDK1 * SGK3 /(1 + ki_rt_014 * ppRB) - vm_rt_016 * pSGK3
rt16a	GSK3 → pGSK3	kc2_rt_016a * pAkt * GSK3 - vm_rt_016a * pGSK3

rt17	mTORC1 → amTORC1	mTORC1 * (kc2_rt_017a * pAkt + kc2_rt_017b * pSGK1)*(1 + alpha_rt_017 * K4Da) - vm_rt_017 * amTORC1
rt18	FOXO3 → pFOXO3	FOXO3 * (kc2_rt_018a * pAkt + kc2_rt_018b * pSGK1) - vm_rt_018 * pFOXO3
rt19	S6K → pS6K	kc2_rt_019 * S6K * amTORC1 - vm_rt_019 * pS6K
rt20	S6 → pS6	S6 * (kc2_rt_020a * pS6K) - vm_rt_020 * pS6
rt21	dRas → tRas	dRas *(kc2_rt_021a * pHER3L + kc2_rt_021b * IRp /(1 + Ki_rt_004 * pS6K) + kc2_rt_021c * mGABp)/(1 + Ki_rt_021 * pErk) - vm_rt_021 * tRas
rt22	Raf → aaRaf	kc2_rt_022 * tRas * Raf / (Km_rt_022 + Raf) / (1 + Ki_rt_022 * pAkt) - kc2_rt_022 * aaRaf
rt23	Mek → pMek	kc2_rt_023 * (1 + alpha_rt_023 * tRac1) * aaRaf * Mek / (Km_rt_023 + Mek) - vm_rt_023 * pMek
rt24	Erk → pErk	kc2_rt_024 * Erk * pMek - vm_rt_024 * pErk
rt25	→ SGK3	vs_rt_025 * (ER + pER) /(Km_rt_025 + (ER + pER))
rt26	SGK3 → ∅	kdeg_rt_026 * SGK3
rt27	∅ → NDRG1	vs_rt_027
rt28	NDRG1 → ∅	(vs_rt_027/50) * NDRG1
rt29	NDRG1 → pNDRG1	kc2_rt_029*NDRG1 * (pSGK1 + pSGK3) - vm_rt_029 * pNDRG1
rt30	pNDRG1 → ∅	kdeg_rt_030 * pNDRG1 * GSK3 / (Km_rt_030 + pNDRG1)
rt31	∅ → HER3	vs_rt_031 * FOXO3/(Km_rt_031 + FOXO3)
rt32	HER3 → ∅	deg_rt_031 * HER3 * (1 + alpha_rt_031 * (NDRG1 + pNDRG1))
rt33	HER3 + HRG → HER3L	ka_rt_033 * HER3 * HRG * HRG_on - kd_rt_033 * HER3L
rt34	HER3L → pHER3L	kc1_rt_034 * HER3L - vm_rt_034 * pHER3L
rt34a	pHER3L → iHER3L	kc1_rt_034_a * pHER3L * (1 + alpha_rt_034_a * pErk) - vm_rt_034_a * iHER3L
rt35	∅ → ER	vs_rt_035b * FOXO3/(Km_rt_035 + FOXO3)
rt36	ER → ∅	deg_rt_035 * ER
rt37	ER → pER	kc2_rt_037 * pErk * ER - vm_rt_037 * pER
cc01	∅ → RB	vs_cc_001
cc02	RB → ∅	(vs_cc_001/100)*RB
cc03	RB → pRB	kc1_cc_003 * RB * (K4Da + K4Da_p21) - vm_cc_003 * pRB
cc05	pRB → ppRB	kc2_cc_005 * K2Ea * pRB - vm_cc_005 * ppRB
cc07	∅ → E2F	tr_cc_007a * E2F/(Km_cc_007b + E2F) + tr_cc_007b * (cMyc /(Km_cc_007a + cMyc)) * (E2F /(Km_cc_007b + E2F)) + tr_cc_007c*(cMyc /(Km_cc_007a+cMyc))

cc08	E2F → ∅	E2F *(kdeg_cc_008b + kdeg_cc_008a * K2Ea)/(Km_cc_008 + E2F)
cc09	E2F + RB → E2FRB	(ka_cc_009 * E2F * RB - kd_cc_009 * E2FRB)
cc10	∅ → Cd	vs_cc_010c * amTORC1 /(Km_cc_010b + amTORC1)
cc11	Cd → ∅	kdeg_cc_011 * Cd *(1 + kc2_cc_011 * GSK3/(Km_cc_011 + GSK3))
cc12	Cd + CDK4 → K4D	ka_cc_012 * Cd * CDK4 - kd_cc_012 * K4D
cc13	K4D → K4Da	kc1_cc_013 * K4D - vm_cc_013b * K4Da
cc13a	K4Da → CDK4	kc2_cc_013a * K4Da * GSK3 /(Km_cc_011 + GSK3)
cc14	K4Da + p21 → K4Da_p21	ka_cc_014 * K4Da * p21 - kd_cc_014 * K4Da_p21
cc14a	K4Da_p21 → CDK4	kc2_cc_014a * K4Da_p21 * GSK3 /(Km_cc_011 + GSK3)
c15	∅ → Ce	vs_cc_015 * E2F /(Km_cc_015 + E2F)
cc16	Ce → ∅	kdeg_cc_015 * Ce
cc17	Ce + CDK2 → K2E	ka_cc_017 * Ce * CDK2 - kd_cc_017 * K2E
cc18	K2E → K2Ea	kc1_cc_018a * K2E - vm_cc_018b * K2Ea
cc19	K2Ea + p21 → K2Ea_p21	ka_cc_019 * K2Ea * p21 - kd_cc_019 * K2Ea_p21
cc20	∅ → p21	vs_cc_020 * pErk /(Km_cc_020 + pErk)
cc21	p21 → ∅	p21 * (deg_cc_021 + kc2_cc_021b * K2Ea / (Km_cc_021b + K2Ea))
cc22	p21 → p_p21	kc2_cc_021a * pAkt * p21/(Km_cc_021a + pAkt)
cc23	p_p21 → ∅	p_p21*(deg_cc_021/10 + kc2_cc_021b * K2Ea / (Km_cc_021b + K2Ea))
cc25	∅ → cMyc	vs_cc_025a * amTORC1 /(Km_cc_025a + amTORC1) + vs_cc_025b * (ER + pER) /(Km_cc_025b + (ER + pER))
cc26	cMyc → ∅	deg_cc_025 * cMyc * (1 + kc1_cc_026 * GSK3)

Supplementary Table 2.

Ordinary differential equations (ODE) of the integrated PI3K signaling network model

(Reaction rates are included in Supplementary Table 1).

**Left-hand Sides**	**Right-hand Sides**
[IGF]/dt	- rt01
[IR]/dt	-rt01
[IRL]/dt	+rt01- rt02
[IRp]/dt	+rt02 - rt03
[IRi]/dt	+rt03
[PI3Ka]/dt	-rt04 - rt06
[PI3Kb]/dt	-rt05 - rt07
[IRp_PI3Ka]/dt	+rt04
[IRp_PI3Kb]/dt	+rt05
[pHER3L_PI3Ka]/dt	+rt06
[pHER3L_PI3Kb]/dt	+rt07
[PIP3]/dt	+rt08 - rt09
[PIP2]/dt	-rt08 + rt09
[PDK1]/dt	-rt11
[mPDK1]/dt	+rt11
[GAB]/dt	-rt10
[mGABp]/dt	+rt10
[PREX1]/dt	-rt12
[mPREX1]/dt	+rt12
[dRac1]/dt	-rt13
[tRac1]/dt	+rt13
[Akt]/dt	-rt14
[pAkt]/dt	+rt14
[SGK1]/dt	-rt15
[pSGK1]/dt	+rt15
[SGK3]/dt	-rt16 + rt25 - rt26
[pSGK3]/dt	+rt16
[GSK3]/dt	-rt16a
[pGSK3]/dt	+rt16a
[mTORC1]/dt	-rt17
[amTORC1]/dt	+rt17
[FOXO3]/dt	-rt18
[pFOXO3]/dt	+rt18
[NDRG1]/dt	+rt27 - rt28 - rt29

[pNDRG1]/dt	+rt29 - rt30
[S6K]/dt	-rt19
[[pS6K]/dt	+rt19
[S6]/dt	-rt20
[pS6]/dt	+rt20
[dRas]/dt	-rt21
[tRas]/dt	+rt21
[Raf]/dt	-rt22
[aaRaf]/dt	+rt22
[Mek]/dt	-rt23
[pMek]/dt	+rt23
[Erk]/dt	-rt24
[pErk]/dt	+rt24
[HER3]/dt	+rt31 - rt32 - rt33
[HRG]/dt	-rt33
[HER3L]/dt	+rt33 - rt34
[pHER3L]/dt	+rt34 - rt34a
[iHER3L]/dt	+rt34a
[ER]/dt	+rt35 - rt36 - rt37
[pER]/dt	+rt37
[RB]/dt	+cc01 - cc02 - cc03 - cc09
[pRB]/dt	+cc03 - cc05
[ppRB]/dt	+cc05
[E2F]/dt	+cc07 - cc08 - cc09
[E2FRB]/dt	+cc09
[Cd]/dt	+cc10 - cc11 - cc12
[CDK4]/dt	-cc12 + cc13a + cc14a
[K4D]/dt	+cc12 - cc13
[K4Da]/dt	+cc13 - cc13a - cc14
[Ce]/dt	+cc15 - cc16 - cc17
[CDK2]/dt	-cc17
[K2E]/dt	+cc17 - cc18
[K2Ea]/dt	+cc18 - cc19
[p21]/dt	-cc14 - cc19 + cc20 - cc21 - cc22
[K4Da_p21]/dt	+cc14 - cc14a
[K2Ea_p21]/dt	+cc19
[cMyc]/dt	+cc25 - cc26
[p_p21]/dt	+cc22 - cc23

Supplementary Table 3.

List of antibodies, small molecule inhibitors, primers and guideRNAs

MATERIALS	SOURCE	IDENTIFIER
Antibodies		
Rabbit monoclonal anti-Phospho-Akt (Ser473) (clone D9E)	Cell Signaling Technology	Cat# 4060; RRID:AB_2315049
Rabbit monoclonal anti-Phospho-Akt (Thr308) (clone D25E6)	Cell Signaling Technology	Cat# 13038; RRID:AB_2629447
Rabbit monoclonal anti- Phospho-S6 Ribosomal Protein (Ser240/244) (clone D68F8)	Cell Signaling Technology	Cat# 5364, RRID:AB_10694233
Rabbit monoclonal anti-S6 Ribosomal Protein (clone 5G10)	Cell Signaling Technology	Cat# 2217; RRID:AB_331355
Mouse monoclonal anti-Actin (clone AC-40)	Sigma-Aldrich	Cat# A3853; RRID:AB_262137
Rabbit monoclonal anti-Phospho-4E-BP1 (Thr37/46) (236B4)	Cell Signaling Technology	Cat# 2855; RRID:AB_560835
Rabbit monoclonal anti-Akt (pan) (clone 11E7)	Cell Signaling Technology	Cat# 4685; RRID:AB_2225340
Rabbit monoclonal anti-Phospho Rb (Ser807/811)	Cell Signaling Technology	Cat# 8516; RRID:AB_11178658
Rabbit monoclonal anti-Phospho-p44/42 MAPK (Erk1/2) (Thr202/Tyr204)	Cell Signaling Technology	Cat# 4370; RRID:AB_2315112
p44/42 MAPK (Erk1/2)	Cell Signaling Technology	Cat# 4696; RRID: AB_390780
Rabbit polyclonal anti-Cyclin D1	Cell Signaling Technology	Cat# 2922; RRID:AB_2228523
Mouse monoclonal anti-Cyclin A (clone AT10.2)	Santa Cruz Biotechnology	Cat# sc-53227; RRID:AB_782329
Rabbit monoclonal anti-CDK4 (clone D9G3E)	Cell Signaling Technology	Cat# 12790; RRID:AB_2631166
Mouse monoclonal anti-CDK6 (clone DCS22)	Cell Signaling Technology	Cat# 3136; RRID:AB_2229289
Rabbit monoclonal anti-CDK2 (clone 78B2)	Cell Signaling Technology	Cat# 2546; RRID:AB_2276129
Rabbit monoclonal anti-p21 Waf1/Cip1 (clone 12D1)	Cell Signaling Technology	Cat# 2947 RRID:AB_823586

Mouse monoclonal anti-p18 INK4C (clone DCS118)	Cell Signaling Technology	Cat# 2896; RRID:AB_331203
Rabbit monoclonal anti-p27 Kip1 (clone D69C12)	Cell Signaling Technology	Cat# 3686; RRID:AB_2077850
Rabbit monoclonal anti-Phospho-Chk1 (Ser345) (clone 133D3)	Cell Signaling Technology	Cat# 2348; RRID:AB_331212
Mouse monoclonal anti-PCNA (clone PC10)	Cell Signaling Technology	Cat#2586; RRID:AB_2160343
Rabbit monoclonal anti-CDKN2A/p16INK4a (clone EPR1473)	Abcam	Cat# ab108349; RRID:AB_10858268
Mouse monoclonal anti-p53 (clone 1C12)	Cell Signaling Technology	Cat# 2524; RRID:AB_331743
Rabbit polyclonal anti-53BP1	Abcam	Cat# ab21083 RRID:AB_722496
Mouse monoclonal anti-phospho-Histone H2A.X (Ser139) (clone JBW301)	Millipore	Cat# 05-636, RRID:AB_309864
Rabbit monoclonal anti-phospho-NDRG1 (Thr346) (D98G11) XP®	Cell Signaling Technology	Cat# 5482; RRID:AB_10693451
Rabbit monoclonal anti-hospho-SGK3 (Thr320) (D30E6)	Cell Signaling Technology	Cat# 5642; RRID: AB_10694357
Small Molecule inhibitors		
BYL719 (Alpelisib), PI3Kalpha inhibitor	Selleck Chemicals	Cat# S2814; CAS:1217486-61-7
MK-8776, Chk1 inhibitor	AdooQ Bioscience	Cat# SCH 900776; CAS: 891494-63-6
GSK2334470, PDK1 inhibitor	Selleck Chemicals	Cat # S7087; CAS 1227911-45-6
**Quantitative PCR**		
Human CDKN1A<br>FWD:TGTCCGTCAGAACCCATGC<br>REV: AAAGTCGAAGTTCCATCGCTC	PrimerBank – MGH-PGA	PrimerBank ID: 310832423c1
CRISPR reagents and crRNA sequences		
Hs.Cas9.TP53.1.AA<br>/AltR1/rCrC rArUrU rGrUrU rCrArA rUrArU rCrGrU rCrCrG rGrUrU rUrUrA rGrArG rCrUrA rUrGrC rU/AltR2/	Integrated DNA Technologies (IDT)	N/A
Hs.Cas9.CDKN1A AB<br>/AltR1/rCrUrUrUrGrUrCrArCrCrGrArGrArCrArC rCrArCrGrUrUrUrUrArGrArGrCrUrArUrGrCrU/AltR2/	Integrated DNA Technologies (IDT)	N/A
Hs.Cas9.CDKN1A AD<br>/AltR1/rCrCrArArGrCrUrCrUrArCrCrUrUrCrCrCrArCrGrGrUrUrUrUrArGrArGrCrUrArUrGrCrU/AltR2/	IDT	N/A
Alt-R® CRISPR-Cas9 Negative Control crRNA #1, 2 nmol	IDT	Cat# 1072544

Alt-R® CRISPR-Cas9 Negative Control crRNA #2, 2 nmol	IDT	Cat#1072545
Alt-R CRISPR-cas9. tracrRNA-5'ATTO 5nmol	IDT	Cat# #1075934
Alt-R S.p. Cas9 Nuclease 3NLS	IDT	Cat#1074181
Nuclease Free Duplex Buffer 2ml	IDT	Cat# 1072570
Opti-MEM™ Reduced Serum Medium, GlutaMAX™ Supplement	Thermofisher Scientific	Cat# 51985034
Lipofectamine 3000	Thermofisher Scientific	Cat# L3000-008

Supplementary Table 4.

Ordinary differential equations (ODE) of the phenotypic model. CC: Cell Cycle signals (= cyclin D1 + cyclin E, outputs of the PI3K network model). G1: G1-phase, S: S-phase, A: Apoptosis, R: DNA repair, M: G2/M-phase. DD: DNA damage signal caused by PI3Kα inhibitor BYL719. DD_0: the basal DNA damage signal that naturally occurs due to various internal and external factors but not directly caused by specific external agents such as BYL719. pCHK1: phosphorylated and activated CHK1 by DD. MK-8876: CHK1 inhibitor. The kinetic parameters and constants used for the simulation of the phenotypic model are provided in **Supplementary Dataset 3**.

**Left-hand Sides**	**Right-hand Sides**
d[G1]/dt	= -kc_g1_s·G1·CC + kr_s_g1·S
d[S]/dt	= kc_g1_s·G1·CC - kr_s_g1·S – kc_s_m·(1/(1+Ki_chk1·pCHK1) + kc_dd·DD /(1+Ki_chk1·pCHK1) + kc_chk1·pCHK1)·S
d[A]/dt	= kc_dd·DD·S /(1+Ki_chk1·pCHK1)
d[R]/dt	= kc_chk1·pCHK1·S – kc_r_m·R
d[M]/dt	= kc_s_m·S/(1+Ki_chk1·pCHK1) + kc_r_m·R
	DD = kc_byl·(DD_0 + BYL719/Ki_byl)<br>pCHK1 = kc_chk_dd·DD/(1 + MK8876)<br>CC = cyclin D1 + cyclin E (outputs of the mechanistic model)
