# Supplementary material for: Integrative modeling uncovers p21-driven drug resistance and prioritizes therapies for PIK3CA-mutant breast cancer
Source: NPJ Precis Oncol. 2024 Jan 26;8:20. doi: 10.1038/s41698-024-00496-y (PMC10810864; doi:10.1038/s41698-024-00496-y)
Supplement: Supplementary file 5 — REPORTING SUMMARY [file 41698_2024_496_MOESM5_ESM.pdf]

nature portfolio

Corresponding author(s): DAPR NPJPRECISIONONCOLOGY-02612R

Last updated by author(s): Dec 5, 2023

Reporting Summary

Nature Portfolio wishes to improve the reproducibility of the work that we publish. This form provides structure for consistency and transparency in reporting. For further information on Nature Portfolio policies, see our Editorial Policies and the Editorial Policy Checklist.

Statistics

For all statistical analyses, confirm that the following items are present in the figure legend, table legend, main text, or Methods section.

n/a	Confirmed	
☐	☒	The exact sample size (*n*) for each experimental group/condition, given as a discrete number and unit of measurement
☐	☒	A statement on whether measurements were taken from distinct samples or whether the same sample was measured repeatedly
☐	☒	The statistical test(s) used AND whether they are one- or two-sided<br>*Only common tests should be described solely by name; describe more complex techniques in the Methods section.*
☒	☐	A description of all covariates tested
☒	☐	A description of any assumptions or corrections, such as tests of normality and adjustment for multiple comparisons
☐	☒	A full description of the statistical parameters including central tendency (e.g. means) or other basic estimates (e.g. regression coefficient) AND variation (e.g. standard deviation) or associated estimates of uncertainty (e.g. confidence intervals)
☐	☒	For null hypothesis testing, the test statistic (e.g. *F*, *t*, *r*) with confidence intervals, effect sizes, degrees of freedom and *P* value noted<br>*Give P values as exact values whenever suitable.*
☒	☐	For Bayesian analysis, information on the choice of priors and Markov chain Monte Carlo settings
☒	☐	For hierarchical and complex designs, identification of the appropriate level for tests and full reporting of outcomes
☒	☐	Estimates of effect sizes (e.g. Cohen's *d*, Pearson's *r*), indicating how they were calculated

Our web collection on statistics for biologists contains articles on many of the points above.

Software and code

Policy information about availability of computer code

Data collection	All codes used for modelling have been deposited in Github and can be accessed at https://github.com/NguyenLabNetworkModeling/Integrated-PI3K-Signaling-Network-model, and the SUNDIALS suite (SUite of Nonlinear and DIfferential/ALgebraic equation Solvers, https://computing.llnl.gov/projects/sundials). Patient datasets were collected from the cBioPortal. Protien identification for phosphoproteomics was performed using Uniprot. Proteomics data have been deposited to the ProteomeXchange Consortium via PRIDE [1] partner repository with the dataset identifier PXD033956.
Data analysis	MATLAB, Maxquant v1.5.5.1., Perseus, Ingenuity Pathway Analysis (IPA), CellProfiler, Fiji, Bio-Rad CFX Manager v3.1, FlowJo 10.3.0

For manuscripts utilizing custom algorithms or software that are central to the research but not yet described in published literature, software must be made available to editors and reviewers. We strongly encourage code deposition in a community repository (e.g. GitHub). See the Nature Portfolio guidelines for submitting code & software for further information.

Data

Policy information about availability of data

All manuscripts must include a data availability statement. This statement should provide the following information, where applicable:

- Accession codes, unique identifiers, or web links for publicly available datasets
- A description of any restrictions on data availability
- For clinical datasets or third party data, please ensure that the statement adheres to our policy

Proteomics data have been deposited to the ProteomeXchange Consortium via PRIDE [1] partner repository with the dataset identifier PXD033956. They are publicly available.

Research involving human participants, their data, or biological material

Policy information about studies with human participants or human data. See also policy information about sex, gender (identity/presentation), and sexual orientation and race, ethnicity and racism.

Reporting on sex and gender	N/A
Reporting on race, ethnicity, or other socially relevant groupings	N/A
Population characteristics	N/A
Recruitment	N/A
Ethics oversight	N/A

Note that full information on the approval of the study protocol must also be provided in the manuscript.

Field-specific reporting

Please select the one below that is the best fit for your research. If you are not sure, read the appropriate sections before making your selection.

☒ Life sciences ☐ Behavioural & social sciences ☐ Ecological, evolutionary & environmental sciences

For a reference copy of the document with all sections, see nature.com/documents/nr-reporting-summary-flat.pdf

Life sciences study design

All studies must disclose on these points even when the disclosure is negative.

Sample size	3 independent T47D and Saos2 BYL719- resistant pools of cells were generated to guarantee reproducibility of biological outcomes in independent samples.
Data exclusions	No data were excluded from the analyses.
Replication	All biological assays have been repeated a minimum of 3 times and results showed similar outcomes.
Randomization	Samples treatments were not randomized but assays were repeated a minimum of 3 times by at least 2 independent investigators.
Blinding	Blinding was not used in these studies because the phenotypes of treated cells very consistently showed the same significant outcomes.

Reporting for specific materials, systems and methods

We require information from authors about some types of materials, experimental systems and methods used in many studies. Here, indicate whether each material, system or method listed is relevant to your study. If you are not sure if a list item applies to your research, read the appropriate section before selecting a response.

Materials & experimental systems

n/a	Involved in the study
☐	☒ Antibodies
☐	☒ Eukaryotic cell lines
☒	☐ Palaeontology and archaeology
☒	☐ Animals and other organisms
☐	☒ Clinical data
☒	☐ Dual use research of concern
☒	☐ Plants

Methods

n/a	Involved in the study
☒	☐ ChIP-seq
☐	☒ Flow cytometry
☒	☐ MRI-based neuroimaging

Antibodies

Antibodies used	All reagents and antibodies used in these experiments have been listed in Supplementary Table 3 with their catalog numbers, dilutions, and indication of vendors.
Validation	All antibodies used in these studies were validated by the manufacturers.

Eukaryotic cell lines

Policy information about cell lines and Sex and Gender in Research

Cell line source(s)	T47D, female, ATCC<br>Saos2, female, a gift from Lee Wong.
Authentication	None of the cell lines were authenticated
Mycoplasma contamination	Cell lines were seldomly tested for cytoplasma contamination and resulted negative.
Commonly misidentified lines<br>(See ICLAC register)	No misidentified cell lines were used in these studies.

Clinical data

Policy information about clinical studies

All manuscripts should comply with the ICMJE guidelines for publication of clinical research and a completed CONSORT checklist must be included with all submissions.

Clinical trial registration	*Provide the trial registration number from ClinicalTrials.gov or an equivalent agency.*
Study protocol	*Note where the full trial protocol can be accessed OR if not available, explain why.*
Data collection	*Describe the settings and locales of data collection, noting the time periods of recruitment and data collection.*
Outcomes	*Describe how you pre-defined primary and secondary outcome measures and how you assessed these measures.*

Flow Cytometry

Plots

Confirm that:

☒ The axis labels state the marker and fluorochrome used (e.g. CD4-FITC).

☒ The axis scales are clearly visible. Include numbers along axes only for bottom left plot of group (a 'group' is an analysis of identical markers).

☒ All plots are contour plots with outliers or pseudocolor plots.

☒ A numerical value for number of cells or percentage (with statistics) is provided.

Methodology

Sample preparation	T47D cells, commercially available cell line.
Instrument	BD LSRFortessaTM X-20 flow cytometer
Software	FlowJo 10.3.0 software package

Cell population abundance

Describe the abundance of the relevant cell populations within post-sort fractions, providing details on the purity of the samples and how it was determined.

Gating strategy

Describe the gating strategy used for all relevant experiments, specifying the preliminary FSC/SSC gates of the starting cell population, indicating where boundaries between "positive" and "negative" staining cell populations are defined.

☒ Tick this box to confirm that a figure exemplifying the gating strategy is provided in the Supplementary Information.
